# Water Repellent Coating in Textile, Paper and Bioplastic Polymers: A Comprehensive Review

**DOI:** 10.3390/polym16192790

**Published:** 2024-10-01

**Authors:** Nattadon Rungruangkitkrai, Phannaphat Phromphen, Nawarat Chartvivatpornchai, Atcharawan Srisa, Yeyen Laorenza, Phanwipa Wongphan, Nathdanai Harnkarnsujarit

**Affiliations:** 1 Department of Textile Science, Faculty of Agro-Industry, Kasetsart University, 50 Ngam Wong Wan Rd., Latyao, Chatuchak, Bangkok 10900, Thailand; fagitdr@ku.ac.th (N.R.); phannaphat.ph@ku.th (P.P.); nawarat.char@ku.th (N.C.); 2 Department of Packaging and Materials Technology, Faculty of Agro-Industry, Kasetsart University, 50 Ngam Wong Wan Rd., Latyao, Chatuchak, Bangkok 10900, Thailand; atcharawan.sri@ku.th (A.S.); yeyen.la@ku.th (Y.L.); phanwipa.w@ku.th (P.W.); 3 Center for Advanced Studies for Agriculture and Food, Kasetsart University, 50 Ngam Wong Wan Rd., Latyao, Chatuchak, Bangkok 10900, Thailand

**Keywords:** water repellent, waterproof, coating, textile, paper, bioplastic

## Abstract

Water-repellent coatings are essential for enhancing the durability and sustainability of textiles, paper, and bioplastic polymers. Despite the growing use of sustainable materials, their inherent hydrophilicity presents significant challenges. This review explores advanced coating technologies to address these issues, focusing on their mechanisms, properties, and applications. By imparting water resistance and repellency, these coatings improve material performance and longevity. The environmental impact and limitations of current coatings are critically assessed, highlighting the need for sustainable solutions. This review identifies key trends and challenges, offering insights into developing water-resistant materials that align with environmental goals while meeting industry demands. Key focus areas include coating mechanisms, techniques, performance evaluation, applications, environmental impact assessment, and the development of sustainable coating solutions. This research contributes to the development of water-resistant materials that meet the demands of modern industries while minimizing environmental impact.

## 1. Introduction

Sustainability is the most popular trend in packaging technology today, driving global social, political, environmental, and economic concerns. Sustainable materials and processes are key to reducing the carbon footprint, and various sectors are increasingly adopting them to decrease the planet’s waste burden. These processes and materials not only support circular systems but also facilitate the implementation of a circular economy [1,2]. Evaluating or developing sustainable packaging is a challenge in the industry due to cost constraints, usage, and performance considerations across production and utilization processes. Sustainable materials can be broadly categorized into natural materials, renewable materials, and non-toxic materials [3]. However, choosing natural or renewable materials comes with limitations, such as potential sourcing issues or limited functionality compared to traditional materials. Natural fibers like cotton, wool, and silk are particularly unsuitable for enduring weather conditions due to their inherent hydrophilicity and structural instability when wet [4,5]. Paper, a popular choice for food and transportation due to its natural origin, biodegradability, and easy availability, suffers from susceptibility to deformation when exposed to moisture or water. This is because its high cellulose content includes abundant hydroxyl groups, making it hydrophilic [6,7]. In addition, bioplastic polymers, derived from biomass resources like chitosan, starch, alginate, proteins, poly (butylene adipate-co-terephthalate) (PBAT), and polylactic acid (PLA), offer biodegradability and renewability. However, most bioplastic polymers are hydrophilic substances, making them susceptible to water absorption [8,9,10]. Making hydrophilic materials more water-resistant is a major challenge in developing materials that allow them to be used in a wider variety of applications.

The development of surface hydrophobicity technology, aiming for water resistance or repellency, can be achieved through various coating methods. These technologies offer the ability to endow solid materials with new functionalities by tailoring their chemical and physical characteristics. This results in improved water resistance, which makes them highly desirable in modern industry [11,12,13,14]. Coatings can offer a wide range of additional functionalities beyond water resistance. These include improved thermal stability, UV protection, increased tear and puncture strength, and even the ability to prevent microbial growth or self-cleaning materials [5,9,12,15]. These advancements make coated materials particularly valuable in the food industry, medicine, and the development of intelligent materials. Several coating methods, such as dipping, spraying, plasma treatment, padding, and bar coating, offer distinct advantages and can be utilized to achieve the desired functionalities [13,16,17,18]. The choice of method depends on the desired properties, material characteristics, and production requirements. Therefore, this review article explores the development of waterproof or water-repellent properties of fabric or textile, paper, and bioplastic polymers and provides guidelines for selecting methods and compounds that are appropriate for use. This research will discuss the principles of water-repellent coating technology, recent advancements, and its potential benefits for improving the functionality and sustainability of water-repellent or waterproof materials.

## 2. Water Repellent Coating in Textile

The global demand for textile coatings from various industries is expected to grow by USD 9.59 billion by 4.93% in 2031 (from USD 6.86 billion in 2022). The growing textile industry, deployment of advanced technology, and the selection of appropriate coating materials (e.g., natural rubber) for specific textile end uses are all driving the increasing popularity of textile coatings. The applications of textile coatings are not only limited to clothing but also various other applications such as automotive, footwear, construction, and apparel industries, as well as medical clothing and fire-protecting & flame-retardant fabrics [19].

### 2.1. Application and Mechanism of Water Repellent in Textile

According to Loghin, et al. [20], the performance of water-repellent clothing depends on two primary factors: (i) subjective variables, reflecting the user’s comfort requirements, and (ii) objective variables, which consider environmental conditions and risks. Based on these factors, water-repellent fabrics can be applied to various clothing types, including conventional wet-weather apparel, sports and leisure garments, and personal protective equipment.

1. Conventional Wet-Weather Clothing

An example of conventional wet-weather clothing is raincoats, along with outdoor sports attire (like fishing, camping, and hiking) that require high levels of waterproofing, durability, cleaning resistance, and visibility.

2. Sports and Leisure Garments

Examples of sports and leisure garments include winter sports apparel, self-ventilating waterproof garments, moisture-control garments, and antifungal waterproof garments. These garments typically require good heat and mass transfer capabilities, humidity absorption and transfer, skin dryness, durability, and low weight.

3. Personal Protective Equipment

Personal protective equipment includes work clothes worn in high-humidity, hazardous powder, microbial suspension, toxic vapor, aerosol, clean room, or sterile work environments. These garments must be impervious to water, chemical reagents, and radioactive contaminants.

Water repellency is a crucial parameter for fabrics, particularly for outdoor clothing, as it protects users from environmental conditions. It refers to the fabric surface’s ability to resist wetting and remove water droplets [21]. However, achieving water repellency can be challenging because most textiles are made from moisture-sensitive materials. Several approaches have been developed to overcome this drawback. These methods involve either hydrophobic fabric modification or coating with water-repellent materials. Water-repellent coating technologies include dip-coating, impregnation, padding, sol-gel, plasma, and spray coating. Water-repellent-based coating materials explored include silane, silicone, polyurethane, fluorochemical, wax, stearic acid, acrylate-based coatings, and even unconventional materials like polyvinyl alcohol, super hydrophobic precipitated calcium carbonate (SHPCC), boric acid, and boron-based coatings.

Different coating materials exhibit distinct water-repellency mechanisms. Paraffin-based coatings are primarily deposited through mechanical incorporation, filling fiber pores and spaces between yarns. Silicone and fluorocarbon products form a thin hydrophobic layer on the fiber surface. Nanoparticles can increase surface roughness, enhancing water hydrophobicity and repellency. Fatty acid resin-based coatings are deposited via chemical reactions with the fiber surface.

**Figure 1:**
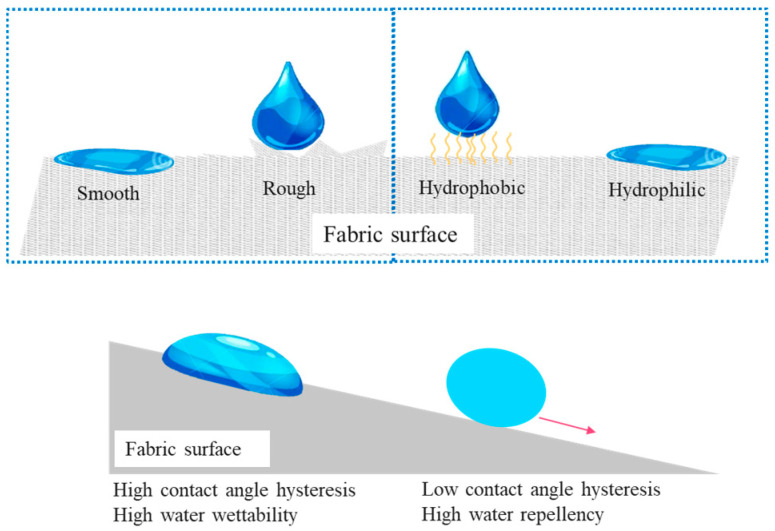
Mechanism of water repellency of rough and hydrophobic fabrics.

The mechanism of water repellency can be explained by the finished coating preventing water droplets from spreading on the fabric surface. To achieve water repellency, the critical surface tension of the coating needs to be lower than the surface tension of water, allowing water to be repelled. Superhydrophobic surfaces exhibit the highest water repellency, with a water contact angle (WCA) exceeding 150° and a water sliding angle (WSA) less than 10° [11,22]. The mechanism of water repellency of fabric is shown in Figure 1.

Water-repellent coatings orient their hydrophobic groups towards the fibers, creating a protective barrier against water. This phenomenon enables water droplets to maintain their spherical shape without spreading or penetrating the fibers, preventing water transfer from the environment. Clothing acts as a barrier, protecting the user from humidity and water droplets.

The water repellency of fabric is determined by its high hydrophobicity and is measured using static water contact angle and contact angle hysteresis [23]. Theoretically, hydrophobic surfaces have a water contact angle (WCA) greater than 90°, indicating reduced attractive forces between water droplets and the fabric surface. WCA can be calculated using Young’s equation:

γSV = γSL + γLV cos θ

where γSV, γSL, and γLV represent the interfacial tension of solid-vapor, solid-liquid, and liquid-vapor, respectively, and θ is the water contact angle at the interface.

In addition to the water contact angle, surface roughness also plays a significant role in hydrophobicity and water repellency. Wenzel’s theory states that liquid droplets may completely penetrate rough cavities. By incorporating the roughness factor ‘r’ into Young’s equation, the relationship between surface tension and water contact angle can be explained:

cosθ* = r cos θ

where r represents the ratio of the actual area to the geometrical surface area, θ* and θ are the external and internal contact angles, respectively. When r is greater than 1, indicating a rough surface, the Wenzel formula is applicable. Besides WCA and CAH, the fabric’s ability to repel water can be simulated using spray tests and rain tests with specific liquids. Table 1 provides a review of water-repellent coated fabrics.

### 2.2. Water Repellent Coating Technology for Textiles

The dip coating technique involves dipping the fabric specimen into the coating solution, followed by solvent removal. The dipping time and cycles significantly affect the amount of coating material deposited on the fabric surface. Silane-based water repellents, such as Hexadecyltri-methoxysilane (HDTMS), Polydimethylsiloxane (PDMS), and alkoxysilanes (3-glycidyloxypropyl)trimethoxysilane, hexadecyltrimethoxysilane, triethoxy(octyl)silane, and triethoxy(ethyl)silane, need to be dissolved in polar solvents like ethanol or isopropanol [11,16,22,24]. Finally, the fabric specimen is dipped into the solution to provide a thin layer of water repellent on the surface.

The spray coating method uses a coating solution similar to the one used in dip coating. The distance between the spray nozzle and the substrate, as well as the number of coating cycles, significantly affect the coating deposition and thickness. Kim, Kim, Song, Cho, Hwang and Chae [21] coated a cotton woven fabric with fluorine, wax, and silicone-based water repellents using spray coating at a distance of 15 cm and repeated the process one, three, and five times. This method provided a uniform coating distribution with a thickness of 3.6 to 4.5 mm. Ke, et al. [25] sprayed a multimodified 2,2,6,6-tetramethylpiperidine 1-oxyl radical (TEMPO)-oxidized cellulose nanofiber (MMT) onto cotton cloth using a spray nozzle at a back pressure of 0.4 MPa for 5 s to achieve a wet density of MMT of 0.5 mL/cm^2^. Celik, Altındal, Gozutok, Ruzi and Onses [17] coated nubuck, denim, chenille, and nonwoven polyester with hydrophobic SiO_2_ coating solution using a spray gun with an inner nozzle diameter of 0.35 mm and compressed air pressure of 4 bar. The distance between the spray and fabric was set at 15 cm to deposit a 23 mg/cm^2^ coating layer on the fabric surface.

The padding method utilizes padder equipment for coating fabrics. This process follows immersion or impregnation of the fabric in a coating solution. Speed, temperature, and pressure are critical parameters that must be carefully controlled during the padding process to ensure consistent results.

Several studies have employed the padding method for various water-repellent coatings. Gargoubi, et al. [26] used this method for fluorochemical coating on cotton fabric with a padding speed of 7 m/min at ambient temperature and a pressure of 1 bar, achieving a pick-up percentage of around 80%. Similarly, Sfameni, et al. [27] coated polyester fabric with alkoxysilanes using a two-roller lab padder with a nip pressure of 2 kg/cm^2^, achieving a pick-up of approximately 70%. Xu, Jiang, Peng, Wang, Shang, Miao and Guo [5] coated at a pressure of 1 kg/m^2^. They varied the cotton fabric with a PVA solution using a laboratory dyeing padder at room temperature with a process of 1–7 cycles to achieve the desired coating thickness. Yu, et al. [28] utilized a vertical two-roll padder to coat cotton with a mixture of cross-linked amino long-chain alkyl polysiloxane (CAHPS) and rSiO_2_. The cotton fabric was immersed in the coating solution for 30 s and then passed through the padder to achieve a pick-up of 65–90%.

**Table 1:** A review on water repellent-based coating technology for textile.

Coating Compounds	Substrate Materials	Coating Method	Coating Solvent	Concentration of Coating Compounds in Solvent	Other Additives	Solvent Removal Method	Major Findings	Minor Finding	Reference
Stearic acid	100% cotton fabric, plain woven fabric	Padding method	Isopropyl alcohol		Citric acid, catalyst sodium hypophosphite (SHP) and enhance triethanolamine (TEA)	Drying at 100 °C for 3 min, and curing at 180 °C for 2 min.	- The fabric coated with stearic acid and citric acid with catalyst SHP and enhancer TEA had the highest water repellency rating of 4 and 5. - The coated fabric without catalyst is wet or readily absorbs the water. Moreover, it had a WCA value ranged from 138° to 145°.	- The interaction between stearic acid, citric acid, and cellulose occurred via ester bond, revealed in FT-IR spectra in 1712 and 1734 cm^−1^ attributed to C=O stretching. - SEM images showed a successful coating deposition onto cotton fabric at yarn and fiber level.	[4]
PVA	Woven cotton fabric	Impregnation and padding method	Liquor	5%	Ag NPs	Drying at 50 °C	- PVA coating causes a sticking between cotton fiber and more compact. While Ag NPs coating showed a fiber remains separated. - The WCA value of uncoated cotton is 0°, it is increased to 120–127° after coating with PVA and Ag NPs. While it is increased by increasing PVA coating cycles.	- The Ag NPs coated cotton causing a decrease in 2θ located at 14.9°, 16.7°, 22.8°, 34.6°. - Ag NPs-PVA coated fabric had lower UV transmittance than uncoated and PVA-coated fabric. - The Ag NPs coated fabric exhibited antimicrobial activity against *Staphylococcus aureus* up to 99.93% after 24 h of contact.	[5]
Ag NPs/ Polydi methyl siloxane (PDMS)	Twill weave fabric (100% cotton)	Dip-coating	Iso-propanol	PDMS (20 and 40 g/L)	Ag NPs (0.2, 0.4, 1, 2, and 4 g/L)	Scouring using non-ionic detergent (2 g/L) at 40 °C for 30 min, followed by drying at room temperature overnight.	- PDMS coating reduces surface roughness. - Increasing amounts of Ag NPs increases the surface roughness. - The WCA increases with increasing surface roughness at higher Ag NP concentrations. - Samples with higher superhydrophobicity exhibit lower contact angle hysteresis (CAH), which in turn leads to a lower sliding angle (SA), indicating better water repellency. - PDMS-40 coated cotton, the CAH increases with increasing Ag NP content.	- PDMS coating increased thermal stability of the fabrics, due to the protective role of PDMS as a heat barrier. - The antibacterial activity is more highly dependent on the concentration of Ag NPs than on superhydrophobicity.	[11]
Super hydrophobic precipitated calcium carbonate (SHPCC)	Polyester fabric	Dip-coating	Stearic acid (SA) and iso-propyl alcohol	4%		Washed with iso-propyl alcohol and then air-dried.	- Higher saturation SHPCC coated fabric exhibited highest WCA 150° which categorized as superhydrophobic surface, while the uncoated fabric directly absorbed the water droplet. - The SA/SHPCC is successfully deposited on the fabric surface, it is intensified the FT-IR peak at 2929 and 2889 cm^−1^ attributed to C-H stretching and C-H bending, respectively.	- The coating causes a microrough on the fabric surface, where SA acts as a binder for SHPCC further enhance hydrophobicity. - The fabric coated with SHPCC showed a Td at 620–720 °C	[16]
Hydrophobic SiO_2_	Nubuck, denim, chenille, and nonwoven polyester	Spray-coating	Ethanol	2%		Drying at room temperature for 1 h.	- The superhydrophobic surface was observed on coated textile with WCA 158–172°. Coated chenille and nonwoven exhibited the highest WCA while nubuck had the lowest WCA. - The sliding angle (SA) of coated textile was around 3°, while nubuck exhibited the highest SA (20°). Lower SA indicates better water repellence ability. - Superhydrophobic chenille and nonwoven fabric were resistant to water spray and water jet impact with value of 200 cycles and 600 s, respectively. While denim and nubuck are susceptible to water impact. Denim had water spray and water jet impact of 5 cycles and 10 s, respectively.	- SEM images showed a waving pattern of uncoated lining and denim, while chenille, nonwoven fabric, and nubuck had a random arrangement. - SiO_2_ deposition on the sample surface was detected in EDX, where O become stronger, and C become weaker after coating.	[17]
Fluorine, silicone, and wax-based water-repellent	Cotton woven fabric	Spray-coating with 15 cm of distance	The water repellent agent is in a liquid state.			Curing at 80 °C for 10 min.	- Wax-based water repellent was the most effective with a water repellency rating of 4, compared to fluorine (1.35) and silicone (2.05)-based water repellent. - The water repellency of the fabric was increased by increasing the coating layer.	- The treated fabric had higher tensile elongation compared to untreated one, as it increased with increasing the coating layer. However, it started to decrease at 5 coating layers assembled.	[21]
Hexadecyltrimethoxysilane (HDTMS) alcosol	80% calcium alginate + 20% polyester (PET)	Dippin	Ethanol	0, 0.3, 1, 3, 5, 7%		Drying at 22 °C for 20 h	- The water droplet cannot stay, and it is permeated through the pristine/uncoated fabric, while 3% HDTMS treatment increased WCA and WSA to 158° and 9.3°, respectively. - Hydrolyzed and condensed HDTMS was successfully introduced on the fabric surface as revealed in FTIR peak. - The pristine fabric was completely wet and swelling deformed (A_s_ = 359.5%) after water immersion for 7 days, HDTMS treated fabric remained dry with A_s_ = 124.1%.	- The 3% HDTMS treatment reduces the breaking strength from 3.84 to 2.4 MPa. - The pristine alginate fabric was smooth and became rough after HDTMS treatment	[22]
Polydimethylsiloxane (PDMS)	PET fabric	Dip-coating	Ethanol	5 g/L		Drying in oven 70 °C for 30 min	- The Si-O-Si peaks at 1200–1000 cm^−1^ attributed to the crosslinking of PDMS on the PET fabric. - The good water repellency of PDMS coated PET fabric was observed by WCA and SA up to 152.2° and 8.4°, respectively. - The water repellency of PET fabric was observed by wetting behavior. PET fabric was totally wet, while PDMS coated PET fabric remained dry after contact with water.	- The uncoated PET fabric had a smooth surface. The PDMS coating creates a uniform thin with some wrinkles on the PET fabric. - The high breathability on pristine PET fabric indicates the presence of open holes which allow the air permeation. The air permeability of PET fabric increases after coating with PDMS, but the water vapor permeability decreases.	[24]
2,2,6,6-tetramethylpiperidine 1-oxyl radical (TEMPO)-oxidized cellulose nanofiber (TOCN), Amidation modified TOCN (AMT) + polyisocyanate cross-linking agent (PCA), Multimodified TOCN (MMT) MMT is the final product for coating material.	Cotton cloth	Spray coating	Tetrahydrofuran (THF)	0.5 wt.%		Drying in vacuum oven at 70 °C for 2 h.	- The TOCN was successfully modified to MMT, revealed at FT-IR spectra at 2274 and 1700 cm^−1^ indication of presence of isocyanate group and carbamate linkage between isocyanate and hydroxyl group, respectively. - MMT coated cotton exhibited a superhydrophobic with WCA~151°, the value was close to commercial fluorine-based water repellence. - The combination of MMT coating and surface roughness plays an important role in the superhydrophobicity. - The WCA was slightly reduced to 125° after 10 washing cycles. The rolling angle was increased by increasing washing cycle.	- SEM images showed that AMT coating cannot adhere well on the cotton surface and showed an aggregate. The MMT coating had better coating ability due to carbamate linkage between isocyanate hydroxyl group.	[25]
Long chain and short chain fluorochemical	Cotton fabric	Impregnation, padding and curing method		Long chain (3 and 5%), short chain (10 and 30%)		Drying at 90 °C for 20 min, followed by curing 1 and 5 min at 120 °C and 150 °C	- The cotton fabric coated with short and long chain fluorochemical had a similar WCA value (130°), indicates the short and long chain fluorochemical would have similar result by modifying its concentration. - The water repellence of the coated cotton fabric was evaluated by spray rating method. It is shown that all the coated cotton had high water repellence (up to 90°), which increase by increasing fluorochemical concentration. - The fluorochemical coating results in dense structure, causing less air permeability but increase shear stiffness.	- The overlapped peaks of F-C-F and C-O-C of the fluorochemical appeared between 1100 and 1250 cm^−1^. - ATR spectrum showed a new peak 1740 cm^−1^ in short-chain fluoropolymer. The peak is correspondent to ester moieties in polyacrylate.	[26]
3-glycidyloxy(propyl)trimethoxysilane (G), hexadecyltrimethoxysilane (C16), triethoxy(octyl)silane (C8) and triethoxy(ethyl)silane (C2)	Plain weave PET fabric	Dip-Pad-Dry-Cure Method	Ethanol				- The highest WCA was achieved by the coated sample PL-G-C2-C16 and PL-G-C8-C16 with WCA value of 150.17° and 147.43°, respectively. While the PL-G (control) had WCA value of 140.32°. - The spray test water repellency showed that the longer alkyl chain of alkoxysilanes, the better ability to repel the water. PL-G had a rating number of 50, while long chain alkyl coated PL-G-C8-C8 and PL-G-C16-C16 exhibited rating numbers up to 100.	- Nano scale surface of treated fabric observed by SEM images, support the hydrophobicity. - The air permeability of the treated fabric was slightly reduced, compared to pristine fabric.	[27]
Hexamethyldisiloxane (HMDSO)	Cotton fabric	Plasma polymerization				Washing with ethanol and acetone	- The treated cotton fabric had WCA higher than 150° and water shedding angle were close to 10° - The ATR-FTIR showed a non-significant change between before and after 10 cycles of washing. However, the intensity was slightly decreased indicating a decreasing of coating polymer due to partial removal during washing. This also effects on decreasing the water repellency ability.		[29]
Fluoropolymer	Toray-Toteron Cotton (TC) is made of 65% polyester and 35% cotton.	Spray coating			TiO_2_ as antimicrobial coating via immersion	Drying at room temperature, for 24 h. Finalization by heat treatment using iron.	- TiO_2_ coating results in rough surface. - The water repellency of unwashed coated fabric remained stable after 60 min of water contact. However, the stability was reduced after ~10 cycles of washing. On the other hand, the uncoated fabric had 0 WCA value which means no water repellency and instantly absorbs the water droplet.	- The uncoated fabric and fluoropolymer coated showed no inhibition effect, while TiO_2_ coated fabric showed an inhibition zone against *Staphylococcus aureus* and *Klebsiella pneumoniae* of 4.9 and 6.3 mm, respectively. - The coated fabric had higher mechanical properties, explained by the coating material fill the interstices at the fiber-fiber region of spun yarn.	[30]
Organosilane-modified cellulose nanofibers	Cotton fabric	Immersion		0.25, 0.5, and 0.75% *w*/*v*		Drying and curing at 120 °C for 2 h, followed by immersion in acetone solution for 24 h to remove unreacted silane. Finally, fabric was dried at 60 °C	- Organosilane deposition results in rough surface of the cotton fabric. It also reduced air and water permeability. - Uncoated fabric had a hydrophilic property as the water absorbed instantly. - WCA and water absorption time increased as the concentration organosilane modified cellulose coating increase	- The coated cotton fabric exhibited antimicrobial activity against *Staphylococcus aureus* and *Escherichia coli*. The effectiveness was reduced after 15 cycles of washing. - A grafting of organosilane in the cellulose chain was revealed in FTIR spectra peak at 1000 and 1200 cm^−1^ attributed to Si-O-Si and Si-O-C, respectively.	[31]
Boric acid or boron-based coating	Cotton fabric	Sol-gel impregnation	Ethanol	Molar ratio of 0.1, 0.5, 1 and 2.5	TiO_2_ dissolved in ethanol, hydrochloric acid, and water with molar ratios of 0.5, 60, 0.008, and 55, respectively.	Drying at 80 °C for 1 h, and curing at 120 °C for 1 h	- The FTIR peak located at 800 cm^−1^ attributed to Ti-O-Ti and Ti-C stretching vibration of TiO_2_ particle was increased, indicates a successful TiO_2_ deposition on the cotton fabric. - The uncoated cotton instantly absorbs the dropped water, while the coated cotton could hold the water on the fabric surface until 600 s after water dropping. - The water repellency was increased by increasing molar ratio of boric acid.	- A significant agglomeration on fabric surface was observed on the TiO_2_-boron-coated cotton fabric. - The coating increased char formation of cotton detected on the T_g_ = 600 °C. - The coating process reduced tensile strength of the cotton fabric due to clogging between yarn on the fabric.	[32]
rSiO_2_ and cross-linked amino long-chain alkyl polysiloxane (CAHPS)	Cotton fabric	Immersion and padding method	Deionized water	20–120 g/L	Emulsifier: isomeric alcohol ethoxylates	Dry at 100 °C for 3 min and cure at 150–170 °C for 60–180 s.	- The coated cotton fabric had a good water repellency. - The contact angle of drops of salt water, tea, coffee, dying solution, milk and cola on the coated fabric were 147.8°, 148.5°, 148.3°, 150.2°, 145.9°, and 148.6°, respectively. - The uncoated cotton fiber is moistened after contact with water. While the coated cotton fiber could maintain its dryness. - The WCA still remained high at 144.2° and 140.9° after 15 and 30 cycles of washing. Result indicates the strong adhesive bonding between rSiO_2_-CAHPS coating and cotton fiber. - The dry rubbing test was used to evaluate the fabric surface abrasion that could affect on water repellency loss. - The WCA dropped to 137.6° and 112.2° after 5 and 20 rounds of dry rubbing.	- The rSiO_2_-CAHPS coated cotton fabric still have a good air permeability, thus it is considering as breathable fabric.	[33]
Polyurethane	Palm fiber/polyester fiber nonwoven composite	Impregnation		5% by weight compared to established nonwoven	Sol-gel treatment using chloropropyl-triethoxylane (CPTS) and tetraethyl orthosilicate (TEOS)	Drying in 120 °C for 20 min	- A nonwoven treated with PU and sol-gel using CPTS (nonwoven-PU-CPTS) showed optimum water repellence using spray and rain test method. - This treatment further reduced water absorption. However, the PU coating alone did not mitigate the water absorption. - Aliphatic chain presence in CPTS creates a barrier against moisture.	- Sol-gel and PU coating reduced elongation at break and increased tensile strength. Indicates the stiffness of the nonwoven was increased after sol-gel treatment and PU coating. FTIR confirmed that condensation capacity of hydrolyzed CPTS is greater than hydrolyzed TEOS.	[34]

**Figure 2:**
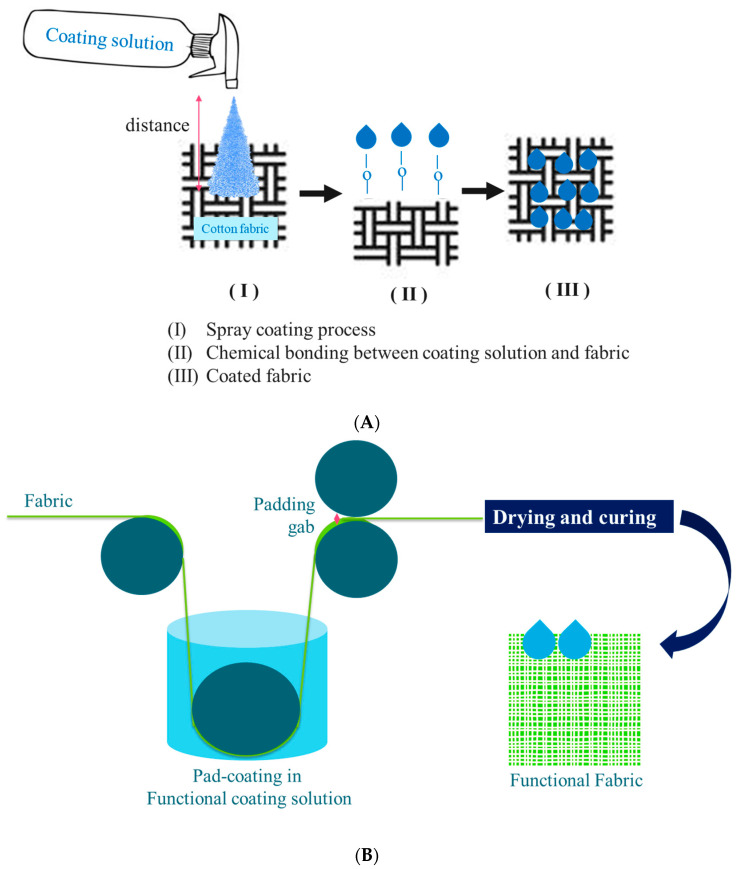
An illustration of (**A**) spray coating and (**B**) pad-coating method for fabric.

The comparison between spray and padding method for coating is shown in Table 2. The coating process is illustrated in Figure 2.

### 2.3. Water Repellent Coating Material for Textile

#### 2.3.1. Fluorochemical

Fluorochemical-based coating materials have been widely used on textiles due to their excellent water repellency. However, they leave behind harmful residual chemicals. These chemicals contain long-chain polymers with perfluoroalkyl acids (PFAAs). When PFAAs degrade in the environment, they release perfluorooctanoic acid (PFOA) and perfluorooctanesulfonate (PFOS), which are toxic to humans, animals, and the environment [26]. The search for alternatives to fluorochemicals has led to the exploration of fluorine-free chemicals and short-chain fluorochemicals. Short-chain fluoropolymers have smaller molecules, making them potentially less toxic in the environment. Gargoubi, Baffoun, Harzallah, Hamdi and Boudokhane [26] compared long-chain and short-chain fluoropolymers, modifying application conditions to achieve similar water-repellency properties for both. They coated cotton fabric with long-chain fluoropolymer (3 and 5 wt.%) and short-chain fluoropolymer (10 and 30 wt.%) and observed similar WCA values (127–135°) for both types. These results indicate that chain length and coating concentration significantly affect the finished textile’s hydrophobicity. Once the surface becomes saturated with the polymer, all pores will be covered. Sharif, et al. [35] explain that long-chain fluorocarbon C8 chemistry has been banned in the textile industry due to its harm to living organisms. Research and industry are currently focusing on short-chain fluorocarbons like C2 and C4, but their stability and durability as water repellents still need improvement. However, the short-chain of PFAs, including perfluorohexane sulfonic acid (PFHxS) and perfluorobutane sulfonic acid (PFBS), has been regulated by US EPA with maximum contaminant level (MCL) of 100 ppt and 1 hazard index, respectively. The future outlook is focused on developing fluorine-free coating materials.

Short-chain PFAAs are often used as substitutes for wax or polish due to their inferior performance compared to long-chain PFAAs. The production process and life cycle of short-chain PFASs used in water-repellent materials release residues into the aquatic and terrestrial environment. As unbound residues, they can easily reach the environment. Short-chain PFAAs are extremely persistent, remaining in the environment for decades.

The residual of short-chain PFAAs has been found in aquatic animals from the Baltic Sea [36]. Guillemot eggs have been found to contain PFBS and PFHxA, while wild boar have been found to contain PFBA. In Svalbard, Norway, PFBA has been found in reindeer. In Ebro, Spain, PFBS, PFBA, and PFHxA have been found in fish. Short-chain PFAAs have also contaminated drinking water due to their high mobility and the limitations of current water treatment technologies [37].

#### 2.3.2. Silane

Hexadecyltrimethoxysilane (HDTMS) is a silane group with a silicon atom bonded to three methoxy groups. It is categorized as a non-fluorinated, low-surface energy alkyl siloxane [22]. When applied to alginate textiles, the HDMS-alcohol coating creates a low surface energy and a rough fiber surface, resulting in a superhydrophobic textile. Studies have reported a WCA of up to 160.8° and WSA < 10° for fabrics treated with HDTMS. This demonstrates that the combination of low surface energy and a rough surface structure contributes to the superhydrophobicity of the alginate textile. The water absorption of alginate textiles is also significantly reduced after coating with 3 wt.% HDTMS. Swelling absorbency (A_s_) is reduced to 124.1% compared to 359.5% for untreated alginate textiles after 7 days of immersion in water.

Alkoxysilanes are another type of silane that has been successfully used as a water-repellent-based coating. Sharif, Mohsin, Ramzan, Ahmad and Qutab [4] investigated alkoxysilanes with different alkyl chain lengths, including triethoxy(ethyl)silane (C2), triethoxy(octyl)silane (C8), and hexadecyltrimethoxysilane (C16). They found that combining short and long hydrocarbon chains, particularly C2-C16 and C8-C16, resulted in high WCA values. This is because the asymmetric nanostructured hyperbranched structures formed from these combinations create a brush effect and surface roughness. Additionally, longer alkyl chains in alkoxysilanes enhance the coated fabric’s ability to repel water. Fabrics coated with C8-C8 and C16-C16 exhibited a repellency rating of 100 in water repellency spray tests, while fabric coated with C2-C2 only achieved a rating of 50. Other silanes successfully used as water-repellent-based coatings include trimethoxy (Octadecyl) silane (OTMS) and mercaptopropyl-triethoxysilane (MPTES).

#### 2.3.3. Silicone

Silicones are another category of water-repellent coatings. Polydimethylsiloxane (PDMS), a silicone with identical terminating methyl groups, offers good mechanical strength, wear resistance, and low surface energy (20–25 mN/m), making it hydrophobic [24]. When applied to PET fabric, PDMS coating resulted in a high-water contact angle (WCA) of up to 152.2° and a high-water sliding angle (WSA) of 8.4°.

Pakdel, Kashi, Sharp and Wang [11] studied the effect of PDMS concentration on cotton fabric. They found that increasing the PDMS concentration from 20% to 40% resulted in a smoother surface due to the binder filling gaps between fibers and yarn. Conversely, incorporating silver nanoparticles (Ag NPs) into the coating solution increased surface roughness, which in turn improved hydrophobicity. The PDMS20/Ag NPs-2 coated cotton fabric exhibited the highest WCA (171.3°). This study demonstrates that a balance between smoothness and roughness can be achieved to optimize water repellency. The contact angle hysteresis (CAH) decreased when the surface roughness increased. The low CAH indicates better water repellency. The PDMS20/Ag NPs-2 coated fabric with a lower CAH exhibited superior water repellency, with water droplets rolling off at a smaller angle.

Hexamethyldisiloxane (HDMSO) is another silicone commonly used for water-repellent coatings. Jebali, Carneiro de Oliveira, Airoudj, Riahi, Fioux, Morlet-Savary, Josien, Ferreira, Roucoules and Bally-Le Gall [29] coated cotton fabric with HDMSO and reported a WCA exceeding 150° and a WSA close to 10°. Notably, the water repellency remained stable even after 10 washing cycles.

Yu, Yang, Yan, Wang, Wang, Qin, Xiong, Shi and Sun [28] investigated a different silicone approach: cross-linked amino long-chain alkyl polysiloxane (CAHPS) combined with rSiO_2_. The hydrophobicity of CAHPS stems from its -C_16_H_33_ chain, while hydroxyl groups can react and form a network with cotton fibers. The presence of silica (rSiO_2_) contributes to a rough surface structure, further enhancing hydrophobicity. Various liquids, including saltwater, tea, dye solution, milk, and cola, were dropped onto the coated fabric surface. All liquids maintained a round shape with a high contact angle up to 147.8°, 148.5°, 148.3°, 150.2°, 145.9°, and 148.6°, respectively. The results demonstrate the fabric’s repellency for various liquids. The water contact angle remained high (around 145°) even after multiple soaping rounds, indicating strong adhesion between the coating and cotton fibers. During the heat curing process, rSiO_2_-CAHPS undergoes an oriented arrangement on the fiber surface. Additionally, the long carbon chain and methyl groups of CAHPS penetrate the fabric surface, while Si-OH and C-OH groups of rSiO_2_-CAHPS bond with C-OH groups of the cotton fabric, further improving water resistance and repellency.

### 2.4. Standard Method for Water Repellency Testing

Several methods are used to evaluate the water-repellency of coated fabrics, including:

Aqueous Liquid Repellency (AATCC Test Method 193-2007): This method uses a common aqueous liquid like water or alcohol. Typically, a 20 µL droplet of the liquid is placed on the fabric surface for 10 s. The highest concentration of the aqueous solution that does not wet the fabric corresponds to a water-repellency rating.

Static Water Contact Angle (ASTM D70354): This method employs the sessile drop technique to measure the contact angle of a 5 µL water droplet on the fabric surface. Fabrics with a WCA greater than 150° are considered good water repellent.

Spray Test (AATCC Test Method 22-2005): In this method, a fabric sample is sprayed with distilled water at a 45° angle from a tester’s funnel. The sample is then knocked three times, and the degree of wetting is evaluated using a water-repellency rating system or a standard spray test rating card.

Rain Test (AATCC Test Method 35-2000): In this, a sample is placed on a weighed blotter. Under controlled conditions, water is sprayed onto the sample for a duration of 5 min. Subsequently, the blotter is reweighed to ascertain the quantity of water that has permeated through the specimen during the testing period.

## 3. Water Repellent Coating in Paper

### 3.1. Paper Coating in Industry

Paper and paperboard are the most popular packaging materials used for several reasons, such as containing and protecting products, providing convenience during storage or consumption, and communicating relevant information to consumers. The global consumption of paper and paperboard totaled 417 million tons in 2021 and is projected to reach 476 million tons by 2032 [38,39]. Paper-based materials hold significant promise for the food packaging industry due to their abundance, affordability, biodegradability, and minimal environmental impact. However, despite these advantages, paper-based materials face limitations for food packaging due to their inherent properties. Paper has limitations in barrier properties, low strength, and heat-seal ability due to pulp fibers containing many hydroxyl groups, which are highly hydrophilic. These hydroxyl groups allow water molecules or moisture to easily penetrate the fibers, reducing their strength by disrupting hydrogen bonding within the pulp structure. In recent years, many researchers have been studying and developing the properties of paper in terms of strength and protection to expand its applications, as shown in Table 3.

### 3.2. Coating Technology for Paper

Dipping and spraying are widely used methods for improving the water resistance of paper due to their convenience, ease of application, and cost-effectiveness. Additionally, these methods often ensure good compatibility between the coating and the paper surface. The Confederation of European Paper Industries (CEPI) reports that in 2021, the use of coated paper reached 17.6%, and this trend will continue to increase, indicating that paper coatings are more effective in commercial applications.

**Figure 3:**
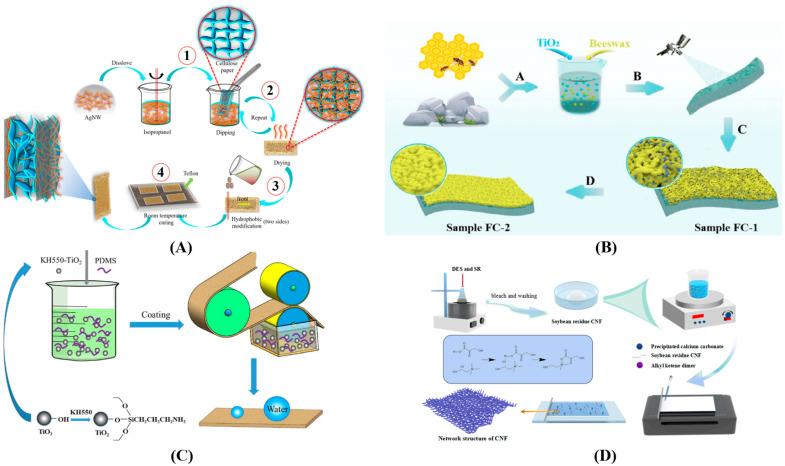
Illustration of coating technology to improve water repellency for cellulose and paper surface using (**A**) dipping process (Reprinted with permission of MDPI Ren, Guo, Guo, Jin, Duan, Ren and Yan [7]), (**B**) spaying (Reprinted with permission of ACS Wan, Xu, Zhu, Li, Wang, Zeng, Li and Chen [12], (**C**) roll coating process (Reprinted with permission of Elsevier Teng, Wang, Shi and Chen [55] and (**D**) iron coating rod (Reprinted with permission of Elsevier Li, Zhou, Jian, Lei, Liu, Zhou, Li, Wang and Zhou [6]).

Figure 3A shows that the dipping process, also known as the immersion method, is an effective technique for surface modification. The resulting properties depend on the type of substrate being treated. In paper coating, for example, the dipping method has been used to enhance paper surfaces by making them more hydrophobic (water-repellent) or waterproof [7]. Organic solutions such as polydimethylsiloxane dissolved in acetone or toluene are commonly employed for this purpose [13,40]. The dipping method offers several advantages, including high efficiency, low cost, and scalability for industrial or commercial applications. Zhang and Zhao [41] demonstrated this by preparing filter paper sheets coated with poly(1-cyanomethyl-3-vinylimidazolium) (a self-crosslinkable polyelectrolyte) using the dipping method. The polyelectrolyte was dissolved in water or dimethylformamide for this process. The results showed that the coated paper surfaces became smoother and exhibited a denser structure with tighter connections between fibers due to the crosslinking effect. Additionally, the coated paper exhibited improved water resistance. Yun, Du, Ji, Tao, Cheng, Lv, Lu and Wang [42] further explored the dipping method by preparing filter paper. Their method involved dipping the paper in a mixed suspension containing ionic sodium carboxymethyl cellulose (a reinforcement agent) for carbon black and multilayer graphene, followed by immersion in silicon dioxide nanoparticles (SiO_2_ NPs) to create a superhydrophobic layer. This approach resulted in coated paper with excellent water stability, superhydrophobicity, self-cleaning properties, and mechanical durability due to the presence of the SiO_2_ NPs.

Spraying is a simple, contactless production method that offers several advantages for coating tear-sensitive materials like paper and fabrics, as shown in Figure 3B. Unlike dipping, spraying allows for more precise control over the coating weight. Additionally, spraying often requires less liquid or solution to achieve the desired results, leading to cost and quality improvements [12,41,45]. Wan, Xu, Zhu, Li, Wang, Zeng, Li and Chen [12] used hydrophilic titanium dioxide (TiO_2_) and hot beeswax emulsion and the coating was sprayed onto the cellulosic paper substrate. The spray coating is easy to prepare, and rapid volatilization results in the formation of a modified surface structure. The coated surface paper was superhydrophobicity after drying and annealing. Ye, Tian, Wang, Ji, Wang and Ci [49] demonstrated the effectiveness of spraying by preparing multifunctional superhydrophobic paper. They used a simple spraying method with a low application amount of 1.5 g/m^2^ of silane-modified superhydrophobic nanofibrillated cellulose. The results showed excellent superhydrophobicity, with a Cobb120s value of only 7.5 g/m^2^, indicating strong waterproof properties. The coated paper also exhibited improved breakage resistance and tear strength due to the formation of bonds between the fibers. Additionally, the nanofibrillated cellulose likely formed stronger hydrogen bonding and van der Waal forces with the fibers, further enhancing its mechanical strength.

In addition, Coating machines offer another set of methods for applying waterproof coatings (Figure 3C,D). These methods include bar coating [6], Mayer coating stick, wire rod coater [43,55], and manual coating with a brush [18,54]. Coating machines are generally convenient, easy to use, and adaptable to a variety of materials, making them well-suited for laboratory applications. By using these methods, researchers can achieve improved surface properties, such as water resistance, waterproof properties, increased durability, and enhanced strength in paper or other materials.

### 3.3. Coating Materials and Applications for Paper

The coatings used are of various types, including bio-based polylactic acid, polyvinyl alcohol, nanoparticle, or cellulose [6,15,41,55]. Sundar, Kumar, Pavithra and Ghosh [15] reported that Kraft paper was coated with a bio-based polylactic acid solution. Bio-based polylactic acid embedded within the cellulose fiber enhances compatibility and adhesion between the coating solution and the paper surface. Moreover, the crystallinity of bio-based polylactic acid improved the interfacial adhesion between the coating solution and Kraft surface, leading to improved abrasive properties. Wang, Xu, Wang, Yang and Zhang [13] used the polyvinyl alcohol (PVA) for base paper coating. PVA-coated paper formed a thin layer that covered the paper surface, improving the water contact angle from 97° to 105° and showing the waterproof performance by reduction of the Cobb values. The Cobb value was determined by the water absorption capacity, indicating that it is water resistant. Moreover, the use of copolymer or mixing solution for coating effectively improved the water resistance performance.

**Figure 4:**
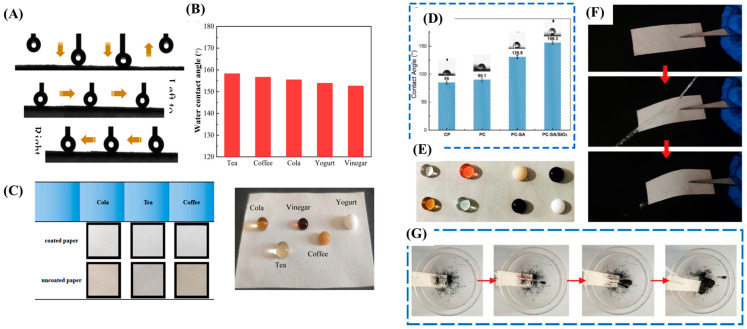
Image of (**A**) dynamic test of adhesion of water droplets to the surface of soybean residue nanocellulose coated waterproof paper, (**B**) water contact angles of different droplets on soybean residue nanocellulose coated paper, (**C**) changes of coated paper and uncoated paper immersed in different liquids and images of liquid drops of waterproof paper in different liquids (Reprinted with permission from Li, Zhou, Jian, Lei, Liu, Zhou, Li, Wang and Zhou [6]), (**D**) water contact angle of cellulose paper (CP), coated paper (PC), coated paper with stearic acid spraying (PC-SA), coated paper with stearic acid/silicon dioxide spraying (PC-SA/SiO_2_), (**E**) the state of different liquids, (**F**) bouncing with the water column and (**G**) self-cleaning property (Reprinted with permission from Wei, Shi, Yao, Zhi, Hu, Yan, Shi, Yu and Huang [56]).

Polydimethylsiloxane (PDMS) is mostly used for coating that can blend with another solution for improving the hydrophobicity due to the molecular chain of PDMS contains a large number of hydrophobic groups in its molecular chain and provides low surface energy, good as well as being environmentally friendly [6,47,55]. In recent years, research has explored improving the barrier and waterproof properties of paper by developing materials with superhydrophobic properties [6,18,42,45,47,49,52]. A superhydrophobic surface has a water contact angle exceeding 150° and a sliding angle less than 10°. This unique characteristic offers a range of properties beneficial for packaging materials, such as antibacterial effects, self-cleaning abilities, corrosion resistance, and antimicrobial properties. Superhydrophobic surfaces often exhibit rough microstructures with low surface energy, similar to the structure of lotus leaves. Moreover, the coated paper with a superhydrophobic surface or with other substances that increased its waterproof properties also affected other properties, such as mechanical strength, thermal resistance, and acid and alkali resistance (Figure 4A–G). Li, Zhou, Jian, Lei, Liu, Zhou, Li, Wang and Zhou [6] investigated the use of soybean residue nanocellulose as a waterproof agent for paper coating. Their research showed that this coating effectively increased the water contact angle to over 150°, indicating superhydrophobicity. The covalent and hydrogen bonds formed between the nanocellulose and the paper’s cellulose fibers resulted in a more compact structure, leading to improved mechanical properties and reduced water absorption. Notably, the coated paper achieved a Cobb value of 14.5 g/m^2^, significantly lower than the standard for food packaging applications (less than 30 g/m^2^). This translates to an 88.2% reduction in water absorption rate. Additionally, the study explored the adhesion resistance of the coating surface using water droplets. They observed that the water droplets left no residue or stains on the paper and adhered to the needle, indicating a weak adhesion force between the water and the coated surface. This further strengthens the paper’s water resistance. The research also measured the water contact angles of various liquids commonly found in food packaging, such as cola, tea, coffee, yogurt, and vinegar. All these liquids exhibited contact angles exceeding 150° on the coated paper, further demonstrating its superhydrophobic properties and potential for diverse liquid food packaging applications.

**Figure 5:**
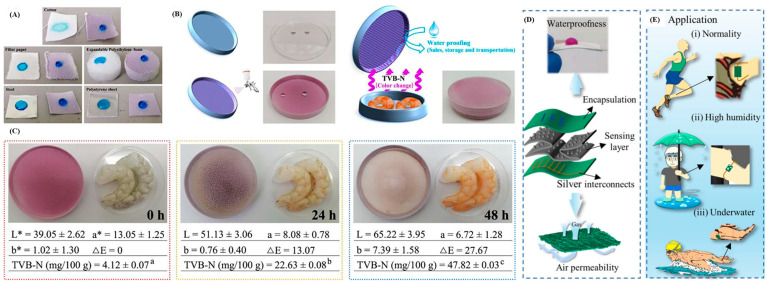
Images of (**A**) water droplets on the coating based on various substrates, (**B**) the coated lid and its application in shrimp freshness monitoring, (**C**) color changing of the coating during freshness monitoring (Reprinted with permission from Wang, et al. [57]), (**D**) illustration for the applications of paper-based coating sensing system and its hydrophobicity and air permeability and (**E**) different application scenarios of the paper-based coating sensing system, including normal, high-humidity and underwater conditions (Reprinted with permission from Wei, Shi, Yao, Zhi, Hu, Yan, Shi, Yu and Huang [56]).

In addition, paper coatings extend their functionality beyond food packaging. Their unique properties make them suitable for developing flexible strain sensors and electromagnetic interference shielding materials, as shown in Figure 5A–E [6,7,42,56].

## 4. Water Repellent Coating in Biopolymers

### 4.1. Bioplastic Substrates

Biopolymers are commonly used as a basis to produce bio-based plastic materials, whether used singly or in blends such as polysaccharides (starch, chitosan, pectin, cellulose, alginate), protein, lipid-derived materials such as poly (butylene adipate-co-terephthalate) (PBAT) and polycaprolactone (PCL) and microbial-derived polymers such as polylactic acid (PLA) [8]. However, biopolymers generally exhibit poor mechanical and barrier properties, especially under high-humidity conditions. This limitation restricts their widespread use in commercial applications. Additionally, forming and stabilizing biopolymer coatings present challenges in improving the material surface’s physical and chemical properties, which are crucial for achieving desired functionalities.

### 4.2. The Biopolymer Coating Materials and Technology

**Figure 6:**
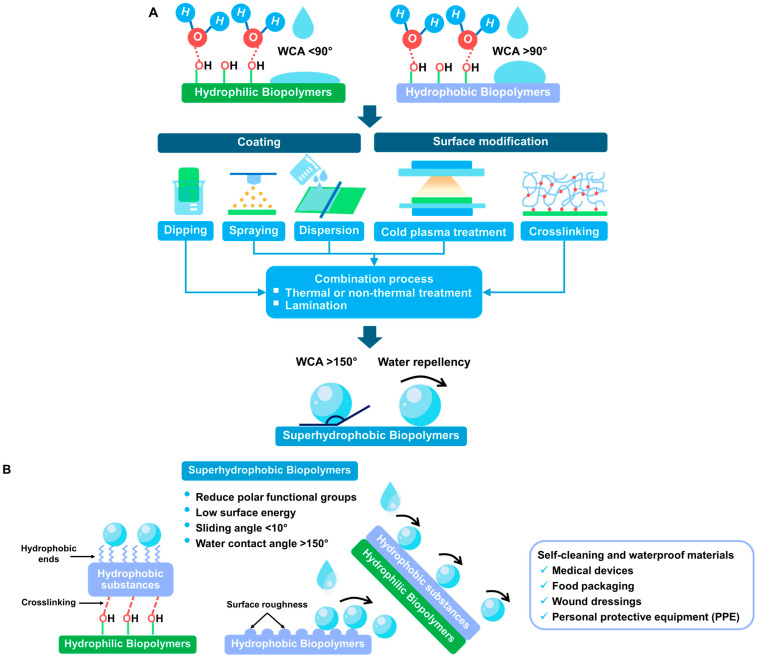
(**A**) Different techniques are used for developing superhydrophobic bioplastics and (**B**) water-repellence mechanisms.

The superhydrophobic biopolymer-based coating can be applied to polymer surfaces, enhancing water repellency, water resistance, water vapor barrier permeability, self-cleaning ability, and resistance to bioadhesion [9,10]. The superhydrophobic effect of these materials is generally indicated by a spherical shape with a high water contact angle (WCA) (>150°), low surface energy, and low sliding angle (<10°) [58]. Fabricating hydrophobic surfaces typically involves a two-step process: (i) building a micro- or nanostructure with a rough surface and (ii) modifying the surface with low surface energy materials [59]. Figure 6A,B demonstrates a common method for producing superhydrophobic bioplastic materials and their water-repellent mechanism. da Fonseca de Albuquerque, et al. [60] employed a surface modification of extruded corn starch films with helium (He) plasma treatment and further coated using hexamethyldisiloxane (HMDSO). The combined He/HMDSO treatment resulted in a more homogeneous with the smoothest surface and smaller grains compared to the sample treated only with HMDSO. He/HMDSO-treated film also exhibits hydrophobic properties, with a WCA exceeding 110°. However, it does not achieve superhydrophobicity (WCA > 150°) due to a decrease in surface roughness.

The two-step process presents limitations for commercial-scale production due to its higher costs, energy consumption, and equipment requirements [59,61]. Chen, et al. [62] developed hydrophobic anisotropic cellulose film by using surface modification via solvent-vaporized crystallization with myristic acid. Increasing the myristic acid concentration to 20% resulted in a hydrophobic surface with the highest WCA (132°) and low surface energy. The H-bonding interaction between myristic acid and cellulose decreases the polar groups of films, resulting in lower water uptake (by 35%) and water vapor permeability (WVP) (by 3.5 × 10^−5^ ± 3.9 × 10^−6^ g·m^−1^·h^−1^·kPa^−1^). In contrast, Wang, et al. [63] fabricated honeycomb coral-like micro/nanostructures of the starch-based absorbent by the hydrolysis-condensation reaction of various methyltrichlorosilane (MTS) concentrations and reaction time. Superhydrophobic adsorbents treated with 6% MTS for 4 h exhibited the lowest surface energy and were identified as the optimal sample due to their high WCA (>151 °C) and low sliding angle (<15 °C). However, these adsorbents exhibited poor durability in violent mechanical treatments, as evidenced by abrasion and tape-peeling tests.

Many researchers have focused on developing low-cost, simple, and sustainable approaches such as dip-coating, solution casting, and spray coating [9,64,65]. The hydrophobicity of the developed film could be influenced by several factors, including the inherent hydrophobicity of the substrate material, modification method (chemical or physical), and coating material. de Souza, et al. [66] employed a dip-coating and heating treatment with a blocked disocyanate solution to enhance the water-repellency of cellulose film. The treatment increased the WCA from 52° to 113° and reduced the water vapor transmission rate (WVTR) from 153 g/m^2^·day to 40 g/m^2^·day (a 74% decrease) for neat film and treated film, respectively. Yu, Yang, Yan, Wang, Wang, Qin, Xiong, Shi and Sun [28] demonstrated ZnO nanoparticle coating and steric acid hydrophobic modification provided the synergy of micro/nano-level hierarchical structure and low surface energy modification. Chitosan film dip-coated with 1% ZnO nanoparticles, and 1% steric acid resulted in excellent superhydrophobicity, evidenced by a maximum WCA of 156°. Additionally, the coated chitosan film demonstrated superior self-cleaning and oil-water separation performance. Recently, Daulay, Ariyanta, Karimah, Santoso, Cahyana, Bukhari, Bakshi, Dungani, Hanifa and Karliati [58] developed superhydrophobic biomedical pulp from rice straw by coating it with a 2:3 mixture of stearic acid and cellulose. The presence of long hydrophobic chains from steric acid on the pulp surface enhanced its superhydrophobic, resulting in a WCA of 153°. Additionally, the treated pulp exhibited improved water resistance for up to 7 days (25 °C) and 3 h (100 °C or hot water).

PLA offers a promising alternative as biodegradable material that can serve as hydrophobic plastic substrates for high-value-added applications. Wu and Then [65] fabricated superhydrophobic surface thermoplastic PLA films via dip-coating with graphene/titanium dioxide nanoparticles (TiO_2_ NPs). The coated films exhibited enhanced surface roughness, low surface wettability, and excellent durability, retaining a WCA above 150° even after 24 h of water immersion testing. Superhydrophobicity of the coated film was achieved with a WCA of 164.21 ± 1° at a graphene-to-TiO_2_ ratio of 1:9. Coating and lamination technologies applied to hydrophobic substrates are ideal for achieving enhanced water resistance and excellent moisture/oxygen barrier performance. Chen, et al. [67] employed the casting method to prepare sandwich-structured films. These films consisted of PLA laminated with a coating layer containing cellulose nanocrystals (CNC) mixed with either polyvinyl alcohol (PVA) or kappa-carrageenan. The synergistic effect of the outer PLA films significantly enhances the water resistance of the CNC core layer. This is evident by the WVP reduction of more than 7 times compared to neat CNC, reaching a value below 3000 g·µm·m^−2^ day^−1^ kPa^−1^. Also, the core layer provides excellent oxygen barrier properties, achieving a reduction of more than 70 times compared to a pure PLA film.

### 4.3. Superhydrophobic Coatings with Multifunctional Properties

There is growing interest in the development and investigation of bioplastic superhydrophobic coatings with multifunctional properties, including water repellency, waterproofing, self-cleaning, and anti-fouling/antimicrobial behavior. Biopolymer materials can be coated with a variety of bioplastic materials to achieve a wide range of applications, as demonstrated in Table 4. These coatings are commonly used on biopolymers like starch, cellulose composites, chitosan, and PLA to create materials for diverse applications, including biomedical applications, recyclable materials, food packaging, disposable waterproof aprons or personal protective equipment (PPE) material, fresh vegetable packaging, super anti-wetting starch film to monitor the freshness of aquatic products. Wang, Ma and Tian [63] demonstrated a superhydrophobic starch-based adsorbent with honeycomb coral-like that exhibited excellent water-repellent, self-cleaning, and anti-fouling properties. Notably, the surface remained completely clean even after contamination with both solid and aqueous liquids during testing. This adsorbent demonstrates promising applications in underwater heavy oil removal and oil slick cleanup on water surfaces. It exhibits an oil adsorption capacity of 2.5–7.6 g/g for various organic liquids.

Carnauba wax and beeswax are natural edible and nontoxic substances with low surface energy. Their primary consists of long-chain hydrocarbons, fatty acids, free alcohols, free acids, and esters, promising candidates for developing low-cost superhydrophobic surfaces [14,61]. Carnauba wax-based coating with a hierarchical structure was employed to enhance the hydrophobicity of the chitosan film [14]. This was achieved by increasing the WCA and decreasing the sliding angle. The coated film exhibited excellent self-cleaning properties towards liquid food, as evidenced by low residual rates of honey and yogurt. Wang, et al. [68] used beeswax coating on tomato waste recycled cutin/pectin membrane to produce an edible artificial lotus leaf. The coated membrane exhibited superhydrophobicity, which is evident from its high WCA of 152° and low sliding angle of 3°. Additionally, this membrane showed anti-fouling and maintained superhydrophobicity in various liquid foods, including water, tea, honey, energy drinks, and cola. These combined properties make it a promising candidate for recyclable functional packaging. To achieve superhydrophobicity in starch/cellulose composites, Niu, et al. [69] combined a two-step coating process with polyethyleneimine as the adhesive layer and beeswax as the functionalized layer. The beeswax-based creates a rough surface with low surface energy, resulting in a WCA of 152° and sliding angle of 6°. The coated film showed excellent self-cleaning ability and anti-fouling performance against various liquid drinks, including milk, cola, coffee, and tea.

Chitosan is a deacetylated chitin derivative that is non-toxic, biocompatible, and can be applied as a coating material to create a water-repellent or waterproof [70,71]. Chitosan-based coating materials were applied via dispersion casting directly onto carrageenan/ZnO composite films [71] and pectin films [64]. The chitosan coating enhanced the surface hydrophobicity of both films, as evidenced by the increased WCA of 91.9 ± 0.6° for carrageenan/ZnO and 89.5 ± 0.7° for pectin film. For the pectin film, the coating increased both the swelling degree and WVP. Conversely, for the carrageenan/ZnO composite film, the coating decreased both parameters. This difference can be attributed to the presence of ZnO in the carrageenan film, which reduced the hydrophilic groups available for binding with water molecules, leading to decreased swelling and WVP.

A multifunctional coating material was developed to improve water repellency, antimicrobial activity, and mechanical strength of the bioplastic substrate. Recent research has focused on developing multifunctional coating materials that can simultaneously improve the water repellency, antimicrobial activity, and mechanical strength of bioplastic substrates. For example, Wang, Qiu and Tian [10] developed super anti-wetting colorimeter starch film by surface modification with a nano-starch/poly-(dimethylsiloxane) (PDMS) composite coating. The synergistic effect of the hierarchical micro/nanostructure formed by nano-starch aggregates and the low surface energy imparted by PDMS contributes to the film’s superhydrophobicity. This is evident from the high WCA of 152.46° and the low sliding angle of 8.15°. The obtained film exhibited self-cleaning properties and repelled various liquid food products, including energy drinks, cola, tea, and honey. Furthermore, this film could be potentially applied to monitor the freshness of aquatic products for 48 h without being disabled by water. Saleh, Kim, Baek and Cha [9] employed spray coating to deposit chitosan-functionalized silica nanoparticles onto both PLA and cellulose-reinforced polybutylene adipate terephthalate (rPBAT) film. As the molecular weight (MW) of chitosan increased, the WCA and water repellency of all films also increased. The chitosan-functionalized silica nanoparticles also had a possible binding affinity to surface polymer chains that improved the fracture strength of all films. Furthermore, the cationic structure of chitosan promotes antibacterial activity against *Escherichia coli*, with up to 90% inhibition for all films. Similarly, Hasanah, Revianashar, Suhendar, Abidin and Pradiva [70] employed spray coating to deposit chitosan crosslinked with glutaraldehyde onto PLA/ZnO/palm wax film for environmentally friendly personal protective equipment (PPE). The chitosan-glutaraldehyde-coated film exhibited significantly lower WVP (37% reduction) compared to the uncoated film. This suggests a near water-repellent or waterproof character based on the permeability analysis. However, increasing chitosan content resulted in increased tensile strength but decreased elongation at the break due to the enhanced Schiff base reaction between chitosan and glutaraldehyde. The highest chitosan content (1.25% *w*/*v*) exhibited greater antibacterial activity against *Escherichia coli* (clear zone 3.2 mm) than *Staphylococcus aureus* (clear zone 2.2 mm). Ye, et al. [72] modified hydroxypropyl methylcellulose (HPMC) films by coating with a composite of polydimethylsiloxane (PDMS) and ball-milled rice starch, corn starch, or potato starch. The synergistic combination of PDMS-coated HPMC with 2 h of ball-milled corn starch resulted in a highly superhydrophobic surface, exhibiting a high WCA of 170.5° and a minimal sliding angle of 5.2°. PDMS/ball-milled starch coatings also exhibit self-cleaning against various food liquids (water, milk, Coca-Cola, and honey) and resistance to *Escherichia coli* adhesion.

## 5. Conclusions and Future Perspectives

This study comprehensively investigates technologies and processes for creating water-repellent surfaces for textiles, paper, and biomaterials. By delving deeper into the ability to modify solid materials to have new properties through novel chemical and physical modifications, this research aims to develop innovative water-repellent coatings. This article emphasizes the development of sustainable coatings, the optimization of coating processes, and the exploration of novel coating materials by adjusting the properties of materials to achieve enhanced water resistance, a critical property for modern industries. Coating technologies using suitable chemical compounds and methods to create coatings pose challenges for various materials. New compounds such as PDMS, nanoparticles, and natural substances like beeswax, chitosan, and cellulose have been employed to enhance material surface properties, making surfaces extremely water-resistant. Materials coated through dipping or spraying can be applied to food packaging with antibacterial and self-cleaning properties or in medical cardiac monitoring, thereby improving the performance and durability of textiles, paper, and bioplastics. Suggestion: notable materials and technologies that have significantly advanced the field of water-repellent coatings, including silicon-based coatings, beeswax, chotisan, nanomaterials, and fluorochemicals such as Polytetrafluoroethylene (PTFE) perfluorooctane sulfonate (PFOS) for water repellency and durability enhancing [73,74,75,76]. Coating technologies enhancing water repellency are gaining prominence as innovations that add functionality and durability to materials. As technology continues to evolve, the future prospects for these coatings are promising, including

▪ Enhanced durability and performance; the development of coatings that are durable, even under harsh conditions, will be crucial.
▪ Coatings that can withstand mechanical stress, such as friction and abrasion, will be essential for applications like outdoor textiles and industrial materials.
▪ Coatings capable of resisting degradation from exposure to various chemicals, including acids, bases, and solvents, will expand their applicability in diverse environments.
▪ The demand for sustainable materials will drive the development of water-repellent coatings derived from renewable resources and that are biodegradable or have minimal environmental impact.
▪ Efforts to minimize the use of harmful chemicals in coating formulations will be a priority to promote sustainability and well-being.
▪ Coatings that can actively repel dirt and contaminants, leading to self-cleaning surfaces, will be highly sought after in various applications, including textiles, building materials, and medical devices.
▪ Incorporating antimicrobial agents into water-repellent coatings can improve antimicrobial properties, making them ideal for applications in healthcare, food packaging, and consumer products.
▪ Coatings that can shield materials from harmful UV radiation will be valuable for applications exposed to sunlight, such as outdoor textiles and solar panels.
▪ Water-repellent coatings can protect electronic components and sensors used in wearable devices from moisture and sweat, enhancing their reliability and durability.
▪ Coatings can improve the performance and longevity of batteries and other energy storage devices by preventing moisture-related degradation.
▪ Water-repellent coatings can enhance the biocompatibility and functionality of medical devices, such as catheters and implants.
▪ The use of nanotechnology can enable the creation of coatings with tailored properties, such as superhydrophobicity and self-healing capabilities.
▪ 3D printing techniques can be used to integrate water-repellent coatings directly into manufactured

Based on these recent findings, the selection of sustainable coatings and materials will be increasingly investigated for future packaging and medical applications. Moreover, A water repellent can be prepared from a water-based emulsion of PDMS. The sample is dipped in the water-based PDMS solution to create an adhesive anchoring layer, then dipped in silica suspension to increase surface roughness, and finally dipped in water-based PDMS emulsion for low surface tension [77]. Polysiloxane water repellents can also be prepared by emulsion with the addition of coupling agents such as 3-glycidyloxypropyltrimethoxysilane (KH560) and gamma-methacryloxypropytrimethoxysilane (KH570). The result showed that a coupling agent ratio of KH570:KH560 = 0.02 mol:0.04 mol had the best water-repellency performance, demonstrating a synergistic effect between the chemical structure and physical morphology. The coating was rougher and more uniform [78]. The DOWSIL™ 520 Dilutable Water Repellent Emulsion is a commercially available water repellent prepared from a silane/siloxane emulsion blend. Therefore, global researchers will explore functional materials and improve coating stability and performance to develop technologies that enhance water resistance and optimize performance. By addressing these future prospects, researchers and industry professionals can continue to innovate and expand the applications of water-repellent coatings, meeting the evolving needs of various sectors and contributing to a more sustainable and technologically advanced future.

## Figures and Tables

**Table 2:** Comparison between spray and padding processes for water repellent coating on textile.

Parameter	Coating Technology	
	Padding Method	Spray Coating Method
Coating material	Liquid coating, primarily based on polymer or resin.	Coating material in liquid, paste, or powder.
Surface Requirement	Generally flat surface.	Can accommodate both flat and uneven surfaces.
Major process	The fabric passes through the roller that contains coating solution.	Fine mist coating material is sprayed onto fabric surface.
Thickness control	Adjusted by controlling the gap between the rollers and using a doctor blade.	Controlled by spray times, spray duration, and nozzle-to-fabric distance.
Drying technology	Tension drying, oven drying, or hot air drying.	Forced air drying, infrared drying, or oven drying.
Advantages	High efficiency for large-scale production, lower equipment costs.	Applicable to a wider range of coating materials, easier control of coating thickness.
Disadvantages	Difficult to control shearing force on textile surface due to large number of process parameter. Consuming a great quantity of solution due to high porosity of the fabric. Longer production time for drying and curing Limited coating types.	Potential for overspray, environmental concerns, higher safety requirements.

**Table 3:** A review on water repellent-based coating technology for paper.

Coating Compounds	Substrate Materials	Coating Method	Coating Solvent	Concentration of Coating Compounds in Solvent	Other Additives	Solvent Removal Method	Major Findings	Minor Finding	Reference
Soybean residue nanocellulose (CNF)	Filter paper	Using the coater with iron coating rod	1.5% Alkyl ketene dimer (AKD) lotion	0.3 wt.% CNF and 0.1 wt.% AKD	2 wt.% of precipitated calcium carbonate (PCC) as a binder	Drying	- CNF-AKD-PCC (CAP) coated paper showed excellent resistance to water droplet adhesion, leading to super hydrophobicity. - Contact angle over 150° for Cola, tea, coffee, vinegar and yogurt droplets, providing excellent liquid resistance. - Coated CAP paper maintained WCA beyond 150° for increasing the folding times, storage temperature, storage days and liquid pH, exhibiting great cold, heat, acid and alkali resistance and stability at storage days. - CAP coated paper reduced Cobb test value 8 times and water absorption rate by 88.2%.	- CAP coated paper improved surface roughness. - CAP coated paper improved tensile strength and elongation at break by 50.3% and 26.3% due to covalent bond and hydrogen bond force between CNF and filter paper.	[6]
Silver nanowires (AgNWs) and hydrophobic inorganic ceramic agent (H)	Filter paper	Dipping and Mayer-rod coating	Isopropyl alcohol	AgNW suspension (10 mg/m)		Drying	- H-AgNW/cellulose paper exhibits excellent hydrophobicity with the water droplet increased from 0° to 141°, indicating an excellent waterproofness. - H-AgNW/cellulose paper showed an antifouling function in dirty water.	- H-AgNW/cellulose paper maintained mechanical flexibility and resistance in comparison to conventional mechanics. - H-AgNW/cellulose paper maintained superior adhesion, even after the peeling off test for 1000 cycles.	[7]
Beeswax and titanium dioxide (TiO_2_)	Cellulosic paper	Spraying	Anhydrous ethanol	0.8 g TiO_2_, 5 g beeswax and 50 mL anhydrous ethanol		Drying and annealing	- The water contact angle of unannealed and annealed samples are all above 150°. - The coated paper showed rough structure and low surface Energy from beeswax. - The coated paper showed resistance to sandpaper-induced wear that showed water contact angle above 147° after four times of friction.	- Thermal annealing had a positive effect on improving the bonding strength between the coating and the paper substrate. - The coated paper showed excellent anti-adhesion properties for liquids (Cola, juice, milk, yogurt and lactobacillus beverage).	[12]
Polydimethylsiloxane (PDMS) and polyvinyl alcohol (PVA)	Paper (70 g/m^2^)	Mayer coating stick and dipping	Water and acetone	2, 2.5, 3, 3.5, 4% *w*/*v* PVA solution and 2 *w*/*v*% PDMS acetone	Hexamethylene diisocyanate trimer as a crosslinking agent	Drying	- PVA-coated paper slightly increased the contact angle of the paper surface from 97° to 105°. - PVA-PDMS-coated paper showed the contact angle value of 2, 3 and 4% PVA for 124.5°, 139° and 104°, respectively. - PVA-PDMS-coated paper decreased the Cobb values from 28.5 g/m^2^ to 11 g/m^2^, indicating excellent waterproof performance.	- PVA-PDMS had good thermal stability and improved barrier performance.	[13]
Poly lactic acid (PLA)	Kraft paper	Bar coater	Methylene dichloride	5%, 10%, 15%, 20%, and 25% PLA		Drying	- PLA- treated Kraft papers reduced the Cobb value in water absorption by 3 to 8.5 times. - Contact angle values increased with increasing concentrations of PLA coatings from approximately 65° to 80°.	- PLA coated papers exhibited good compatibility possessed between PLA and cellulose fibers, affecting its penetration and adhesion. - PLA coated papers reduced surface roughness. - PLA coated papers enhanced the endurance of the paper material to impact load, depending on PLA concentration. - PLA coated papers increased tensile and bursting strength.	[15]
Microcrystalline wax	Paper (grammage 70 g/m^2^, kit rating 0/12)	Manual coating	Deionized water	The solid content of the mixture at about 30%	Span-80 and Tween-80	Drying	- The Cobb_60_ value of the coated paper reduced 11.3% and increasing coating load to 10 g/m^2^ the Cobb_60_ decreased for 54.3%. - Increased coating load increased the water contact angle to 106.1° due to microcrystalline wax caused waterproof performance, and the larger the coating load provided the better the water resistance capability of the paper.	- The coated paper reduced water vapor permeability by 54.1% and increasing coating load reduced water vapor permeability by 96.7%.	[18]
Polysilsesquioxane nanorods (PSNR)	Cellulosic paper	Scaping (PSNR) and using inkjet printer			Trichloroethylsilane	nonsolvent	- PSNR-paper exhibited water contact angle of 162° that was excellent water repellency due to PSNR-paper showed the low surface energy along with the nanoscale surface roughness. - PSNR-paper maintained superhydrophilicity with contact angle above 150° when tested with acid and alkali solution (HCL, NaOH and toluene). - PSNR-paper with both excellent ink adhesion and outstanding water repellency due to the rapid absorption and complete wetting (contact angle of 0°) of the ink on the paper surface.	- PSNR-paper demonstrated mechanical durability and flexibility due to PSNR-paper maintained its contact angle of above 150° after 20 abrasion cycles and did not affect the contact angle after despite 500 bending. - PSNR-paper showed excellent printability toward widely used inkjet printing techniques and showed water repellency after printing or writing.	[40]
Poly [1-cyanomethyl-3-vinylimidazolium] (PCMVIm)	Filter paper sheet	Dipping	Water	0.2%, 0.4%, 0.6%, 0.8%, 1.2%, 1.6%, 2%, 3%, 4%, 5%, 6%, 7%, 8%, 9%, 10% in weight.		Drying and ammonia vapor annealing	- 1.2 wt.% PCMVIm exhibited water resistance. - 0.6 wt.% PCMVIm was resistanct to high temperature and strong acid. - Water contact angle of PCMVIm maintained stable around 105–112° with increasing the amount of PCMVIm.	- PCMVIm gave smoother surface and filled the gaps between fibers, indicating dense networks. - Tensile strength of dry paper increases from 12.1 to 33.1 MPa and wet paper also increased from 0.6 to 20.7 MPa with increasing PCMVIm (0 wt.% to 30 wt.%).	[41]
Multilayer graphene (MG), carbon black (CB), sodium carboxymethyl cellulose (CMC) and silicon dioxide (SiO_2_)	Filter paper	Dipping	Anhydrous ethanol and deionized water (1:1)	CB/MG/CMC is 15 mg/mL and the mass ratio of CB/MG/CMC is 6/4/5.		Drying	- The water contact angle of CB/MG/CMC increased from 12° to 133° and 156° after dipping for one and three times, respectively, indicating super hydrophobicity. - Superhydrophobic property of CB/MG/CMC/SiO_2_ paper showed hierarchal roughness as a result of nanoparticles. - CB/MG/CMC/SiO_2_ paper possessed excellent stability to water flow at different flow rates. - CB/MG/CMC/SiO_2_ paper showed excellent abrasion resistance due to water contact angle drops less than 5 (from 156° to 153°). - CB/MG/CMC/SiO_2_ paper exhibited conductive stability at water droplets interference. - CB/MG/CMC/SiO_2_ paper showed excellent superhydrophobicity, self-cleaning property, antifouling ability and mechanical durability.	- Surface color of composite paper changed from white to black and paper kept excellent flexibility after coating with CB/MG/CMC.	[42]
Beeswax-chitosan	Paper sheet	Wire rod coater	Acetic acid	1% chitosan, 4% glycerol and 30% beeswax	Tween 80	Drying	- Beeswax-chitosan improved water repellency due to the strong hydrophobicity.	- Beeswax-chitosan gave a higher average tensile index than the uncoated base paper.	[43]
Chitin nanocrystals (ChNCs) and hexadecyltrimethoxysilane (HDTMS)	Filter paper	Dipping	Deionized Water and anhydrous ethanol	0.5, 1, 2, 4 wt.% ChNCs and 1.25, 2.5 and 5 wt% HDTMS		Drying	- The paper surface became hydrophobic for 2% ChNCs and different amounts of HDTMS while water contact angle of 1.25% HDTMS was 128.4°. - ChNCs/HDTMS coating s increased water absorption (wetting behavior) which was up to 150.5% for immersing four days. - Water contact angle was stable above 130° after immersing in acid and alkali solution and explored to UV lamp. - The coated paper had excellent abrasion resistance after suffering from various mechanical forces, including bending, finger-wipe and sandpaper.	- ChNCs/HDTMS presented low-surface-energy compound to the filter paper. - ChNCs/HDTMS filled pores, giving a uniform and smooth surface which significantly decreased arithmetic average roughness from 84.3 nm to 38.0 nm. - The tensile strength of coated paper increased with the increase of ChNCs content. - The coated paper presented excellent self-cleaning and anti-fouling performance. - 2% ChNCs/HDTMS decreased water vapor permeability from 14.96 × 10^−3^ to 11.37 × 10^−3^ g⋅m/m^2^⋅Pa⋅h. - The coated paper enhanced thermal properties. - 2% ChNCs/HDTMS exhibited excellent antibacterial adhesion activity with lower numbers of both *E. coli* and *S. aureus* was over 98%.	[44]
Hydrophobic n-SiO_2_	Kraft paper	Spraying	Ethanol	4% hydrophobic n-SiO_2_	Methyltrimethoxysilane and ammonia	Drying	- n-SiO_2_ coated had a flatter surface, a higher contact angle (151.2°), and a higher roughness than the uncoated due to tight network structure and invisible boundaries. - Hydrophobic n-SiO_2_ exhibited water-resistant on the paper surface.	- The coated paper increased dry tensile strength for 56%. - The coated paper increased wet tensile strength for 2277% due to hydrogen bonding force among micro/nanoscale fibers increased exponentially with higher water retention value. - The water vapor transmission rate of coated paper decreased from 1996.60 to 378.24 g⋅m^−2^⋅day^−1^ due to dense structure on the surface. - Coated paper showed air permeability as low as 0.00317 μm⋅Pa^−1^⋅s^−1^.	[45]
Polyimine (PI)	Paper towel (PT), newspaper (NP), and printing paper (PP)	Dipping	Ethanol	44%, 35%, and 16% of PI for PT, NP and PP, respectively.		Drying	- The water uptakes of NP and PP were significantly lower than the untreated NP which correspond to 6-fold and 2-fold decrease, respectively. - The coated paper showed good waterproof performance and resistance to organic solvents.	- Coated OT, NP and PP incresed tensile strength for 3.7, 8.1 and 1.6 times, respectively. - Coated OT, NP and PP maintained wet mechanical strength for 100, 87 and 14%, respectively.	[46]
Trimethylsiloxy-terminated polydimethylsiloxane (PDMS) And hydrophobic fumed silica (Hf-SiO_2_)	Paper sheet	Dipping	Toluene	0.5% PDMS and 3% Hf-SiO_2_		Drying	- The coated paper significantly increased water contact angle by 155° and rapidly decreased sliding angle from 10° to 1°, providing super hydrophobicity. - The water gain after immersion in water for 24 h reduced from 31.9 g/m^2^ to 10.3 g/m^2^ due to superhydrophobic property and less porous structure. - Coated paper demonstrated antifouling and self-cleaning surfaces.	- The PDMS/Hf-SiO_2_ coated paper enhanced tensile stress from 1.6 MPa to 11.0 MPa due to the PDMS/Hf-SiO_2_ formed more condensed and compact fiber network.	[47]
Nanofibrillated cellulose (NFC)	Base paper	Meyer rod coating	Styrene butadiene (SB) latex	0.10, 0.20, 0.30, 0.40% NFC	Calcium carbonate, kaolin and calcined kaolin (45:40:15) and polyvinyl alcohol (PVA),	Drying	- The coated paper displayed increasing the NFC content (0.3–0.4%) decreased the Cobb value from 27.5 to 24.0 g/m^2^ which NFC reduced the gap size of the coated paper, decreasing the liquid penetration.	- The coated paper increased bursting strength, folding endurance and tensile strength due to NFC enhanced of the binding strength between the coating and base paper. - 0.10%, 0.20%, 0.30% and 0.40% NFC increased air resistance, indicating NFC was likely to reinforce the interaction.	[48]
Nanofibrillated cellulose (NFC)	Commercial paper for food packaging	Spraying	Anhydrous ethanol	100 g anhydrous ethanol: 0.5 g NFC	Tetraethyl orthosilicate, ammonia and acetyl trimethoxysilane	Drying	- WCA of paper exceeded 150° and sliding angle was below 10°, indicating super hydrophobicity. - Waterproofing properties were enhanced by spray amount of 1.5 g/m^2^ due to the micro-nano rough structures and low surface energy on the superhydrophobic surface. - The micro nano rough structure and low surface energy of coated paper showed the self-cleaning properties.	- 2.5 g/m^2^ of spraying volume improved the paper strength and increased breakage resistance and tear indices by 12.94% and 10.17%, respectively. - The coating did not affect the water vapor permeability. - The coated paper provided excellent mechanical (rubbing and bending tests) and chemical (acid, alkali, temperature and UV irradiation) stability. - The coated paper influenced the antimicrobial adhesion performance *Staphylococcus aureus* and *Escherichia coli* which was reduced by 98.64% and 98.09%, respectively due to low surface energy prevented bacteria from stably adhering to the coated paper surface.	[49]
Cellulose nanocrystals (CNCs), octadecylamine (ODA) and polytannic acid (PTA)	Filter paper	Spraying	Ethanol	2% ODA-PTA@CNC ethanol dispersion		Drying	- ODA-PTA@CNC coated paper displayed WCA for 158° with rougher surface from a nanoscale structure. - ODA-PTA@CNCs had low surface energy which came from octadecyl chain and the nanoscale structure of CNC rods. - ODA-PTA@CNC-coated paper showed water-repellent.	- ODA-PTA@CNC-coated paper exhibited tensile strength of about 22.7 MPa at the dry state and reduced to less than 2.5 MPa after being wetted state. - ODA-PTA@CNC coated papers used as semipermeable membranes of moisture-absorbing devices as a result of waterproof properties. - WCA of the ODA-PTA@CNC-coated filter paper slightly decreased with the friction distance (more than 100 cm) and remained stable and greater than 140° after sandpaper rub testing, indicating excellent wear resistance.	[50]
Polyvinyl Acetate (PVC) and Lignin	Filter paper	Spraying	Acetone	10 wt.% PVC and 5, 10, 15 and 20% lignin		Drying	- PVC and lignin enhanced the water-resistance and increased contact angle from 2.1° to 90° due to fact that lignin has a hydrophobic nature.	- Increasing the lignin content improved smoother surface and less pore structureresulted of good interpenetration and interaction between paper and copolymers. - Coated paper significantly increased tensile index and burst resistance with the increase of the lignin content. - Lignin increased adhesion of fiber, leading to inhibit sliding and stretching. - Coated paper decreased air permeability which filled the fiber gaps and reduced porosity.	[51]
Polylactic acid (PLA), Stearic acid (SA), cinnamaldehyde (CIN) and nano-silica (SiO_2_)	Cellulose paper	Coating rod and spraying	Dichloromethane and 90% ethanol	10%PLA, 1 g SA, 2 g SiO_2_	Span80	Drying	- Coated SA paper had contact angle of 130.9° which SA has more clusters, giving rougher surface, increasing the area of air trapping and enhancing hydrophobicity. - PC-SA/SiO_2_ enhanced the contact area with air and decreased the surface energy, indicating hydrophobicity with a contact angle of 156.3°. - PC-SA/SiO_2_ exhibited surface’s excellent self-cleaning capability. - the Cobb value of sample PC-SA/SiO_2_ decreased by 100% in 60–300 s, indicating superhydrophobic modified samples and excellent water resistance.	- SA/SiO_2_ increased degradation temperature from 355 to 360 °C, promoting its thermal stability. - Mechanical strength of PC-SA and PC-SA/SiO_2_ increased by 43.83% and 47.94%, respectively. - Coated PLA/CIN showed smooth surface and compact, indicating the excellent film-forming ability. - Oxygen and water vapor permeability of PC-SA/SiO_2_ was greatly reduced from 500,000 to 57.942 cm^3^/m^2^·day·0.1 MPa and 1802.35 to 206.95 g/m^2^ ·day·Pa, respectively. - PC-SA inhibited *E. coli* and *S. aureus* growth by 99.18 ± 0.23% and 99.31 ± 0.46%, respectively and PC-SA/SiO_2_ inhibited both bacteria reached up to 100%.	[52]
Polystyrene (PS), polyethylene vinyl acetate (PEVA), Carboxymethyl cellulose (CC) and Polyvinyl alcohol (PVOH)	Label paper	Commercia rotary printing press	Deionized water	0.1–4.0% PS, 0.5–9.5% PEVA, 0.25–2% CC and 2–9.5% PVOH.	Epoxysilicone blend	Drying	- The WCA of all treatments slightly increased with increased coating compound which the coated paper required WCA achieve the 90° for anti-adhesive properties. - The PS-, PEVA-, PVOH and CC-coated paper showed WCA value for 100°, 110°, 94° and 94°, respectively.	- PS coated paper showed a rough surface while PEVA and PVOH had smoother surface than PS. - Adhesive tape test showed that PS occurred non-uniformly, PEVA showed very little paper residue remaining adhered on the surface, PVOH were no paper residues, indicating that the siliconization occurred evenly over the entire surface, resulting in low adhesion to the hydrophobic silicone structure.	[53]
Chitosan (CTS), laccase treated chitosan (Lac-CTS) and HP treated chitosan (HP-CTS)	Kraft paper	Brush	2% *v*/*v* acetic acid solution	0.1 mg, 0.2 mg, 0.3 mg, 0.4 mg, 0.5 mg and 0.6 mg in 100 mL acetic acid solution		Drying	- Lac/HP-CTS coated paper showed sizing degree for 331.7 s, indicating enhancement of water barrier property. - CTS and HP-CTS increased the Cobb values from 85.4 g/m^2^ to 110.06 g/m^2^ and 94.72 g/m^2^, respectively while Lac/HP-CTS coated paper was decreased to 48.66 g/m^2^ due to chitosan filled the gaps between cellulose fibers and formed a water-resistant layer.	- CTS, HP-CTS and Lac/ HP-CTS coated papers increased dry tensile index by 204.71%, 176.47% and 107.06%. - CTS, HP-CTS and Lac/ HP-CTS coated papers increased the tear index by 65.74%, 79.46% and 36.37%. - CTS, HP-CTS and Lac/ HP-CTS coated papers increased burst index by 117.69%, 110.20% and 42.86%. - SEM of coated paper presented good adhesion between the coating and cellulosic substrate.	[54]
Polydimethylsiloxane (PDMS), γ-aminopropyltriethoxysilane and Titanium dioxide (TiO_2_)	Filter paper	laboratory roll coater	Absolute ethanol	2 g of polydimethylsiloxane, 20 g of γ-aminopropyltriethoxysilane modified TiO_2_ in 100 g of absolute ethanol and		Drying	- The coated γ-aminopropyltriethoxysilane modified TiO_2_ paper showed hemispherical droplets and were slowly absorb. - Water contact angle of γ-aminopropyltriethoxysilane modified TiO_2_ increased from 39° to 87.9° due to the number of -OH decreased and the number of hydrophobic groups -CH_3_ increased. - Water contact angle of PMDS-γ-aminopropyltriethoxysilane modified TiO_2_ increased from 39° to 154.5°, indicating superhydrophobicity. - PMDS-γ-aminopropyltriethoxysilane modified TiO_2_ remained superhydrophobic surface after peeling for 40 cycles, indicating the abrasion performance.	- The roughness of the coated paper was reduced from 11.2 μm to 10.27 μm. - PMDS-γ-aminopropyltriethoxysilane modified TiO_2_ had good chemical stability in acidic and alkaline solutions and in long-term storage. - PMDS-γ-aminopropyltriethoxysilane modified TiO_2_ showed weaker adhesion between the water droplets, indicating good self-cleaning.	[55]

**Table 4:** A review on water repellent-based coating technology for biopolymers.

Coating Compounds	Substrate Materials	Form of Bioplastic	Coating Method	Coating Solvent	Concentration of Coating Compounds in Solvent	Solvent Removal Method	Major Findings	Minor Finding	Potential Application	Reference
Chitosan functionalized silica nanoparticles (CS-SNP)	PLA and cellulose-reinforced polybutylene adipate terephthalate (rPBAT)	Film	Spray coating	Acetic acid	Silica nanoparticles powder mixed 20 mL chitosan solution (in 10% acetic acid) and coated film with varying M_W_ of chitosan low MW (50,000–190,000 Da), medium MW (200,000–300,000 Da) and high MW (310,000–375,000 Da).	Dried at 80 °C overnight	- As the M_W_ of chitosan increased, the WCA and water repellency of all films also increased. - The WCA was maximal at the intermediate M_W_ of chitosan for all substrates.	- For both PLA and rPBAT films, the fracture strengths and tensile moduli were similarly enhanced by chitosan-functionalized silica nanoparticles. - The coating enhanced antibacterial activity against *Escherichia coli*, with up to 90% inhibition for both PLA and rPBAT.	Antimicrobial coating material	[9]
Poly (dimethyl siloxane) (PDMS) mixed with five common commercial nano-starch, potato starch, maize starch, rice starch, cassava starch, and wheat starch	Starch loaded anthocyanin	Film	Spray coating	Ethyl acetate	Mass ratios of 0/0.6, 0.1/0.5, 0.3/0.3, and 0.5/0.1 starch/PDMS composite coatings	Cured at 80 °C for 2 h	- The synergistic effect of the hierarchical micro/nanostructure formed by nano-starch aggregates and the low surface energy imparted by PDMS exhibited superhydrophobicity with extremely high WCA of 152.46° and low sliding angle of 8.15°. - The increase in the nano-starch content caused a notable increase in WVP, water solubility (at 100 and 25 °C) and moisture content. - Nano-starch/PDMS composite coating exhibited self-cleaning properties and repelled various liquid food products, including energy drinks, cola, tea, and honey.	- Tensile strength and elongation at break significantly increased with increasing nano-starch ratio in PDMS coating. - During freshness monitoring, film coated 0.3/0.3 starch/PDMS composite can be clearly distinguished without being disabled by water throughout freshness monitoring for 48 h.	Super anti-wetting colorimetric starch film to monitor the freshness of aquatic products	[10]
Polydimethylsiloxane (PDMS) and carnauba wax	Chitosan	Film	PDMS spread coating and then carnauba wax-based particles sieve covering the surface	-	1 g carnauba wax in 60 mL TiO_2_ dispersion solution with varying the particle size of carnauba wax (23–48 µm and 48–70 µm)	Cured at 45 °C for 6 h	- The addition of carnauba wax particles creates a hierarchical structure on the film surface, resulting in a higher WCA (>135° at 0 s) and lower sliding angle compared to the uncoated film. - Film coated with a smaller size (23–48 µm) carnauba wax-based particles showed higher hydrophobicity than film coated film the larger size (48–70 µm). The smaller size (23–48 µm) carnauba wax-based particles resulted in higher WCA and lower sliding angle. - The coated film with smaller size (23–48 µm) carnauba wax-based particles exhibited excellent self-cleaning properties with low residue rates of liquid foods (yogurt 3.66% and honey 3.05%).	- Carnauba wax particles with a smaller size range (23–48 µm) created a flatter and more anisotropic surface with a higher density of small gaps and particles distributed on the surface.	Hydrophobic coating materials	[14]
ZnO nanoparticle mixed with stearic acid	Chitosan	Film	Dip-coating	Ethanol	0.1%, 0.5%, and 1% of ZnO nanoparticle and 1% of stearic acid	Dried at 60 °C for 10 min	- At 1% concentration of ZnO nanoparticles and stearic acid exhibited the maximum roughness of the composite film (22.4 nm). - Chitosan film dip-coated with of 1% ZnO nanoparticles and 1% steric acid resulted in excellent superhydrophobicity, evidenced by a maximum WCA of 156°. - The WCA of coated chitosan film underwent little change at pH 4–12, and still retained its superhydrophobic properties. 1% concentration of ZnO nanoparticles and stearic acid coated film demonstrated superior self-cleaning and oil-water separation performance.	- The increase in ZnO concentration increased the film’s tensile strength. - The coating improved the thermal stability of the chitosan films.	Food packaging material, outdoor self-cleaning materials and in oil-water separations	[28]
Stearic acid/cellulose composite	Rice straw	Kidney tray (biomedical pulp product)	Resurfacing	Ethanol	Ethanolic stearic acid mixed with cellulose at a 2:3 (*v*/*v*) ratio	Dried at 90 °C for 24 h	- Coating treatment resulted in a superhydrophobic surface for the biomedical pulp, as evidenced by a WCA of 153° and perfectly spherical water droplets. - Coating treatment improved water resistance up to 7 days at 25 °C and 3 h at 100 °C.	- Coating treatment leads to an increase in the tensile strength, the tensile strain, and tensile energy absorption of the pulp product.	Biomedical applications	[58]
Hexamethyldisiloxane (HMDSO)	Corn starch	Film	He plasma modification		Cathode self-bias voltage −60 V for 20 min and 100 V for 10 min		- The combined He/HMDSO treatment resulted in a more homogeneous with the smoothest surface and smaller grains compared to treated with HMDSO only. - Films coated with HMDSO and He/HMDSO became hydrophobic, showing WCA exceeding 110° but does not achieve superhydrophobicity (WCA > 150°) due to a decrease in surface roughness. - The values of the reduction of absorbed water content were: 78.82 ± 3.91 (HMDSO) and 74.81 ± 3.25 (He/HMDSO), % by weight.	- WVP of untreated, treated HMDSO and HE/HMDSO were 8.64 ± 1.47, 8.49 ± 0.12 and 9.74 ± 1.16 (×10^−10^ g·Pa^−1^·s^−2^·m^−1^), respectively.	Packaging fresh vegetables	[60]
Myristic acid	Anisotropic cellulose	Film	Solvent-vaporized crystallization	Ethanol	1, 3, 5, 10, and 20 g/100 mL	Hot-pressing plates	- Increasing the myristic acid concentration to 20% resulted in a hydrophobic surface with the highest WCA (132°) and low surface energy. - At 10% of myristic acid coating exhibited excellent water-repellent and self-cleaning property against various liquid foods including cola, mango juice, yogurt, milk, soy sauce, and vinegar.	- The coated film exhibited good tensile strength and toughness under both dry (188.7 MPa, 34.4 MJ m^−3^) and humid conditions (119.9 MPa, 28.7 MJ m^−3^). - Myristic coating also reduced low water uptake (by 35%) and WVP (by 3.5 × 10^−5^ ± 3.9 × 10^−6^ g·m^−1^·h^−1^·kPa^−1^).	Self-cleaning and waterproof packaging materials	[62]
Methyl trichlorosilane (MTS)	Starch	Porous starch-based nanofiber film and foam	Facile immersion process	Toluene	MTS concentration and reaction time (hours); 2%–4, 6%–2 and 10%–4)	Dried at 60 °C for 30 min	- The MTS coating provided superhydrophobic starch-based adsorbent with low-surface-energy, honeycomb coral-like micro/nanostructures (high WCA > 151.0°, low sliding angle < 15.0°). - The MSC coating exhibited excellent water repellency, self-cleaning properties against coffee powder, and antifouling properties against methylene blue-dyed water and muddy water. - The coated sample exhibited an oil adsorption capacity of 2.5–7.6 g/g for various organic liquids.	- Superhydrophobic starch-based adsorbent exhibits passable mechanical and chemical durability.	Heavy oil removal underwater and oil slick cleaning from the water surface	[63]
Chitosan	Pectin	Film	Solution casting method	Acetic acid	1.5 wt.% in 1% *v*/*v* of acetic acid	Dried at 30 °C for 72 h	- The chitosan coating enhanced the surface hydrophobicity of pectin films that increased WCA of 89.5 ± 0.7°. - The coating increased both swelling degree, resistance to water solubility and WVP. - Bilayer pectin/chitosan films exhibited slightly higher WVP (2.69 ± 0.13 × 10^−11^ mol/m·s·Pa) compared to the neat pectin film (2.15 ± 0.28 × 10^−11^ mol/m·s·Pa).	- Adding layer of chitosan did not improve the mechanical and oxygen barrier properties of the pectin films.	An inner layer for multilayer packaging for high moisture content products	[64]
Graphene and titanium dioxide (TiO_2_) nanoparticles	PLA	Film	Dip-coating	Hexane	0.1, 0.3, 0.5, 0.7, and 0.9% *w*/*v* of graphene/TiO_2_ coating solution	Cured at 50 °C for 1 h	- The graphene/TiO_2_ coating exhibited enhanced low surface wettability, retaining a WCA above 150° even after 24 h of water immersion testing. - Superhydrophobicity of the coated film was achieved with a WCA of 164.21 ± 1° at a graphene to TiO_2_ ratio of 1:9.	- The graphene/TiO_2_ coating exhibited enhanced surface roughness and excellent durability	Medical devices with self-cleaning properties	[65]
Blocked disocyanate solution	Nanofibrilated cellulose	Film	Dip-coating and then thermal treatment at 170 °C for 10 min.	Anhydrous butyl acetate	6 g of blocked disocyanate solution mixed 0.25 g of zinc octanoate in 100 mL of xylene/butyl acetate (75/25).	Washing and dried at 60 °C for 1 h.	- Coated film increased in the average roughness, the mean square root roughness and the maximum profile height. - The treatment increased the WCA from 52° to 113°.	- Oxygen transmission rate (OTR) was decreased from 15 cm^3^/m^2^/day to 0.1 cm^3^/m^2^/day and WVTR was reduced from 153 g/m^2^·day to 40 g/m^2^·day for neat and treated film, respectively.	Flexible films for packaging	[66]
Cellulose nanocrystals (CNC) mixed with 15 wt.% PVA or kappa-carrageenan	PLA	Film	Solution casting method and laminating	Water	6 and 10 wt. %	Dried at room temperature	- Laminating PLA as the outer layers significantly improves the water resistance of the CNC core layer, evidenced by 7 times decreased in WVP (<3000 g·µm·m^−2^ day^−1^·kPa^−1^) compared to neat CNC film (9603 g·µm·m^−2^ day^−1^·kPa^−1^).	- The CNC core layer provides excellent oxygen barrier properties achieving a reduction of more than 70 times compared to a pure PLA film. - The tensile strength of PLA film laminated with either CNC/PVA or CNC/CG coating does not significantly change. - The tear strength of the CNC film was improved when laminated with PLA films.	Recyclable food barrier packaging	[67]
Beeswax	Cutin/pectin membrane	Membranes	Spray coating	Ethanol	2.5 mg/cm^2^ on one/both sides	Dried at 55 °C for 60 s	- Beeswax coated membrane exhibited superhydrophobicity with WCA at 152 °C and sliding angle at 3°. - Beeswax coated membrane showed anti-fouling against various liquid food products including water, honey, tea, energy drink, cola, yogurt, milk and coffee.	- Combination of coating and heat treatment showed no significant mechanical changes to the uncoated membrane. - Beeswax coated membrane could efficiently block the enzymatic browning of apple after placed at room temperature for 24 h. - The coated bag can be easily dissolved into boiled water, as the beeswax melt at temperature around 62 °C.	Recyclable functional packaging for underwater storage, oxidation blocking, selective release bags	[68]
Polyethyleneimine (PEI) and beeswax	Starch/cellulose composites	Film	Polyethyleneimine (PEI) immersion coating and then beeswax spray coating	Absolute ethanol for PEI and hexane and acetone for beeswax	1.26 wt.% PEI solution and varying sprayed beeswax solution for 1, 3, 6, 9 and 12 times	Annealed at 55 °C for 1 min	- The beeswax-based creates a rough surface with low surface energy, resulting in WCA of 152° and sliding angle of 6°. - Water/moisture absorption of film reduced significantly (>30%) after beeswax functionalization. - The coated film showed excellent self-cleaning ability and anti-fouling performance against various liquid drinks including milk, cola, coffee and tea.	- Tensile strength of the beeswax treated substrate was slightly change (≈22 MPa) compared to untreated, while the elongation at break increased as the humidity increased.	Packaging materials	[69]
Chitosan crosslinked with glutaraldehyde	PLA, ZnO, and palm wax	Film	Spray coating	Acetic acid	0.25%, 0.5%, 0.75%, 1.0%, and 1.25% (*w*/*v*) in 25 mL of 2% acetic acid solution	Dried at room temperature	- The chitosan-glutaraldehyde coated film exhibited significantly lower WVP (37% reduction) compared to the uncoated film that almost water repellent or waterproof.	- Increasing chitosan content resulted in increased tensile strength but decreased elongation at break, due to the enhanced Schiff base reaction between chitosan and glutaraldehyde. - At highest chitosan content (1.25% *w*/*v*) exhibited greater antibacterial activity against *Escherichia coli* (clear zone 3.2 mm) than *Staphylococcus aureus* (clear zone 2.2 mm).	Disposable waterproof aprons or personal protective equipment (PPE) material	[70]
Chitosan	ι-Carrageenan/ZnO	Film	Dispersion casting	Acetic acid	1 g of chitosan in 100 mL of 1% wt. acetic acid	Dried at 50 °C for 7 h	- The WCA of the bilayer film (91.9°) was about 1.3 times higher than that of the neat carrageenan films (68.4°). - Bilayer film had the best swelling resistance (anti-expansion performance) in distilled water (1962.78%) and normal saline (830.30%).	- The tensile strength of bilayer film was 51.49 MPa, 1.5 times more than that of carrageenan/ZnO film (28.9 MPa). - WVP of bilayer film (11.75 × 10^−11^ g·m^−1^·s^−2^·Pa^−1^) was decreased compared to carrageenan/ZnO film (15.17 × 10^−11^ g·m^−1^·s^−2^·Pa^−1^). - The bilayer film exhibited greater antibacterial activity against *Escherichia coli* (clear zone 16.19 ± 1.04 mm) than *Staphylococcus aureus* (clear zone 15.73 ± 1.01 mm).	Food packaging materials and wound dressings	[71]
Polydimethylsiloxane (PDMS) mixed with ball-milled rice starch, corn starch, or potato starch	Hydroxypropyl methylcellulose (HPMC)	Film	Spray coating and immersion	Ethyl acetate	Varying ball-milling process (0, 2, 6, and 8)	Thermally curing at 105 °C for 2 h	- PDMS/rice starch and PDMS/corn starch coatings exhibited superhydrophobic behavior, while PDMS/potato starch coatings failed to achieve superhydrophobicity. - The synergistic combination of PDMS-coated HPMC with 2 h of ball-milled corn starch resulted in a highly superhydrophobic surface, exhibiting a high WCA of 170.5° and a minimal sliding angle of 5.2°.	- PDMS/ball-milled starch coatings exhibit significantly less *Escherichia coli* adhesion. - PDMS/ball-milled starch coatings also exhibit self-cleaning against various food liquids (water, milk, Coca-Cola, and honey).	Food packaging materials	[72]

## Data Availability

Data presented in this study are available on reasonable request from the corresponding author.
